# Effect of Network Architecture on Synchronization and Entrainment Properties of the Circadian Oscillations in the Suprachiasmatic Nucleus

**DOI:** 10.1371/journal.pcbi.1002419

**Published:** 2012-03-08

**Authors:** Marc Hafner, Heinz Koeppl, Didier Gonze

**Affiliations:** 1 Laboratory of Nonlinear Systems, School of Computer and Communication Sciences, Ecole Polytechnique Fédérale de Lausanne, Lausanne, Switzerland; 2 Biomolecular Signaling and Control (BISON) Group, Department of Information Technology and Electrical Engineering, ETH Zurich, Zurich, Switzerland; 3 Laboratoire de Bioinformatique des Génomes et des Réseaux, Faculté des Sciences, Université Libre de Bruxelles, Bruxelles, Belgium; 4 Unité de Chronobiologie Théorique, Faculté des Sciences, Université Libre de Bruxelles, Bruxelles, Belgium; Indiana University, United States of America

## Abstract

In mammals, the suprachiasmatic nucleus (SCN) of the hypothalamus constitutes the central circadian pacemaker. The SCN receives light signals from the retina and controls peripheral circadian clocks (located in the cortex, the pineal gland, the liver, the kidney, the heart, etc.). This hierarchical organization of the circadian system ensures the proper timing of physiological processes. In each SCN neuron, interconnected transcriptional and translational feedback loops enable the circadian expression of the clock genes. Although all the neurons have the same genotype, the oscillations of individual cells are highly heterogeneous in dispersed cell culture: many cells present damped oscillations and the period of the oscillations varies from cell to cell. In addition, the neurotransmitters that ensure the intercellular coupling, and thereby the synchronization of the cellular rhythms, differ between the two main regions of the SCN. In this work, a mathematical model that accounts for this heterogeneous organization of the SCN is presented and used to study the implication of the SCN network topology on synchronization and entrainment properties. The results show that oscillations with larger amplitude can be obtained with scale-free networks, in contrast to random and local connections. Networks with the small-world property such as the scale-free networks used in this work can adapt faster to a delay or advance in the light/dark cycle (jet lag). Interestingly a certain level of cellular heterogeneity is not detrimental to synchronization performances, but on the contrary helps resynchronization after jet lag. When coupling two networks with different topologies that mimic the two regions of the SCN, efficient filtering of pulse-like perturbations in the entrainment pattern is observed. These results suggest that the complex and heterogeneous architecture of the SCN decreases the sensitivity of the network to short entrainment perturbations while, at the same time, improving its adaptation abilities to long term changes.

## Author Summary

In order to adapt to their cycling environment, virtually all living organisms have developed an internal timer, the circadian clock. In mammals, the circadian pacemaker is composed of about 20,000 neurons, called the suprachiasmatic nucleus (SCN) located in the hypothalamus. The SCN receives light signals from the retina and controls peripheral circadian clocks to ensure the proper timing of physiological processes. In each SCN neuron, a genetic regulatory network enables the circadian expression of the clock genes, but individual dynamics are highly heterogeneous in dispersed cell culture: many cells present damped oscillations and the period of the oscillations varies from cell to cell. In addition, the neurotransmitters that ensure the intercellular coupling, and thereby the synchronization of the cellular rhythms, differ between the two main regions of the SCN. We present here a mathematical model that accounts for this heterogeneous organization of the SCN and study the implication of the network topology on synchronization and entrainment properties. Our results show that cellular heterogeneity may help the resynchronization after jet lag and suggest that the complex architecture of the SCN decreases the sensitivity of the network to short entrainment perturbations while, at the same time, improving its adaptation abilities to long term changes.

## Introduction

**Figure 1:**
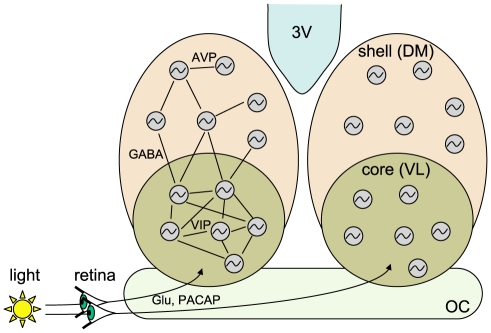
Scheme of the SCN. The SCN is divided in two identical hemispheres (left and right), each composed of two groups of neurons (core and shell, shown on the right hemisphere), distinguished by the type of neurotransmitters they release. In the ventro-lateral part (VL), the neurons mainly express VIP (shown on the left hemisphere), whereas in the dorso-medial part (DM), AVP is expressed. The two parts also differ by their coupling properties. Moreover, only a subset of VL neurons are light-sensitive and are entrained by light cues originating from the optical chiasm (OC).

In mammals, the suprachiasmatic nucleus (SCN) of the hypothalamus constitutes the central circadian pacemaker [1], [2]. The SCN comprises about 20000 densely packed neurons organized into bilateral pairs of nuclei on each side of the third ventricle, above the optic chiasm [2] (Fig. 1). The cells receive light signals from the retina via the optic nerve. The SCN controls circadian rhythms in other parts of the brain including the cortex and the pineal gland, as well as in peripheral tissues such as the liver, kidney, and heart. This hierarchical organization of the circadian system ensures the proper timing of physiological processes and behavior [1], [3]. In natural conditions, the organism is subject to the alternation of days and nights. In response and anticipation to this cycling environment, the circadian pacemaker adjusts the phase of clock-controlled processes with respect to the light-dark cycle.

Each SCN neuron expresses clock genes. Interconnected transcriptional and translational feedback loops form the core circadian network allowing each cell to produce circadian oscillations [4], [5]. Such oscillations still subsist in cultured cells. However, in dispersed culture, the oscillator population is highly heterogeneous: many cells present damped oscillations [6] and the period of the oscillations varies from cell to cell [7]. To produce a reliable global rhythm, the SCN cells must oscillate in synchrony. Synchronization is achieved via intercellular coupling mechanisms [8], [9]. The SCN can thus be regarded as a network of coupled oscillators.

Cells of the SCN can be roughly divided in two groups of neurons that differ by their light sensitivity, the neurostransmitters they produce, and consequently by their coupling properties [2] (Fig. 1). Besides GABA which is expressed by all SCN neurons [10], several region-specific neurotransmitters have been identified. In the ventro-lateral region (VL), the neurons mainly express vasoactive intestinal peptides (VIP), whereas the neurons of the dorso-medial region (DM) express a different neural hormone, the arginine-vasopressin (AVP). When the two regions are dissociated, the VL cells remain synchronized while the DM cells run out of phase [11]. Such results suggest that the two SCN regions differ by their intercellular coupling properties. Additionally, only the VL region is light-sensitive and just a distinct subset of VL neurons is directly influenced by the photic input [12], [13].

Little is known about the connectivity and topological properties of the SCN cellular network. However the characterization of anatomical and functional connectivity in other regions of the brain (e.g. cortex) revealed small-world properties [14], [15], [16]. Small-world topology combines local and long-range connections, thereby decreasing the average path length between cells [17]. Such organization was shown to lead to more efficient synchronization at a lower energy cost (because fewer connections are needed) [18], [19], [20], [21], [22]. It is thus reasonable to assume that the SCN also exploits such network properties to efficiently synchronize neurons.

In this paper we developed a multi-oscillator model for the SCN and investigated the implication of the network topology on synchronization and entrainment properties. The model studied here extends the work previously published by Bernard et al. [23] in three main directions: we introduced heterogeneity among the different SCN cells, we systematically compared generic network topologies, we proposed a model accounting for the distinction between two distinct subareas in the SCN, and investigated the possible role of this separation in the response of the SCN to light signals. The core cellular oscillator is a molecular model of intermediate complexity, which is based on interlocked feedback loops [24]. In the present work, we introduced cellular heterogeneity through variability in parameter values to mimic experimental observations and various topologies for the coupling of the oscillators: random, scale-free, and local networks. Long connections are present in the random and scale-free topologies forming a small-world network [25], [26]. Scale-free networks are characterized by a skewed distribution of the connections where a few cells (hubs) are connected to a large number of cells while the rest have few outgoing edges. On the contrary in a local topology, cells are only connected to their close neighbors. We compared the dynamical properties of the different networks: resynchronization time after a temporary arrest of the oscillations or after a transient decoupling, the synchronization and entrainment performances, as well as the response of the system to jet lags. Finally, we proposed a coupled dual network as a model of the VL-DM organization of the SCN.

## Results

### Model of individual cells

**Figure 2:**
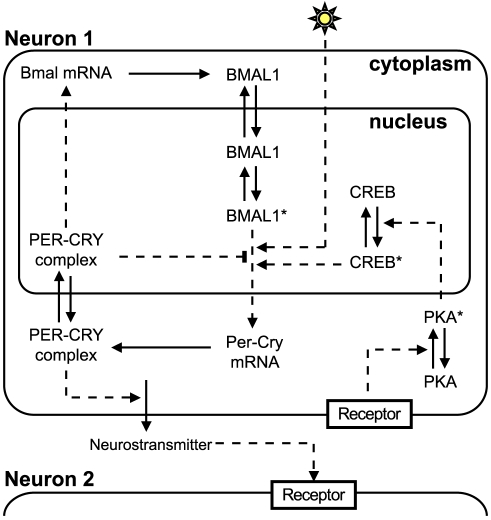
Scheme of the single-cell circadian oscillator. The intracellular oscillator consists of interlocked positive and negative transcriptional/translational feedback loops. In the negative feedback loop, Per and Cry genes (treated as a single variable) inhibit their own transcription by preventing BMAL1 activity. In the positive feedback loop, the PER/CRY complex activates the transcription of their common transcriptional activator, Bmal1 [24]. The release of the neurotransmitter is activated by PER/CRY. In turn, the neurotransmitter activates, in the downstream cell, a signaling cascade (involving PKA and CREB) that increases Per/Cry mRNA expression [23]. In this model, light enhances Per/Cry mRNA expression. In this schematic representation, solid arrows denote transport, translation steps, or phosphorylation/dephosphorylation reactions, while dashed arrows denote transcriptional or post-translational regulations. The stars indicate the active (phosphorylated or complexed) forms of the proteins.

Several models have been proposed for the cellular mammalian circadian clock. Earlier models are mostly phenomenological and rely either on abstract equations [27], [28], or on simple biomolecular mechanisms [29]. More recently, detailed molecular models have been proposed [24], [30], [31], [32]. For our purpose we have chosen the model of intermediary complexity proposed by Becker-Weimann *et al.* [24]. This models does not explicitly incorporate all clock components (for example no distinction is done between Per1–3 and Cry1–2, the Per/Cry complex is denoted by 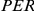), but accounts for the core architecture of the circadian clock, involving interlocked positive and negative feedback loops (Fig. 2). To take into account coupling and light entrainment, the Becker-Weimann model was extended to include a neurotransmitter and a signaling cascade [23]. The coupling between the molecular oscillators is accomplished by a neurotransmitter 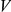, released upon Per/Cry complex activity in the upstream cell. The neurotransmitter triggers, in the target cell, a signaling cascade involving PKA and CREB that have been experimentally shown to activate Per/Cry transcription [33], [34]. The resulting two-step cascade can be seen as a generic signaling pathway. In addition to a modulation by CREB, the production of Per/Cry mRNA (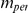) is also increased by light in the light-sensitive cells. Overall, the model we used comprises ten state variables that represent different molecular species or complexes (Fig. 2). Reaction rates are modeled using mass-action kinetics, except for the regulated mRNA production rate where Hill-type functions are used (see Eqs. (1) in *Models*).

**Figure 3:**
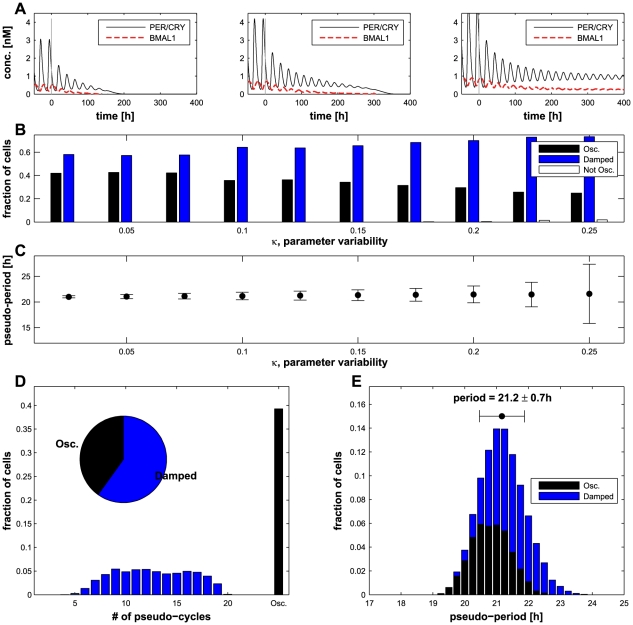
Individual cell variability. (A) Concentration of 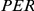 and 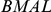 in different cells. The cell is isolated and entrained by light until 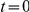, then the number of oscillations (pseudo-cycles) is measured until the relative amplitude is lower than 0.1. (B) Distribution of oscillating and damped cells depending on the parameter variability 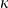. ‘Not Osc.’ stands for ‘Not Oscillating’ (see *Models*). (C) Mean and standard deviation of the pseudo-period of individual cells for different values of 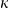. (D) Distribution of the cell dynamics for 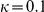: more than one third of the cells show sustained oscillations and the others display from 5 to 20 pseudo-cycles. (E) Distribution of the pseudo-periods for 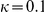 for the oscillating and damped cells (see *Models*).

To mimic the experimentally observed population heterogeneity, we introduce variability in all model parameters except Hill coefficients. For each cell 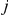, the parameters 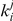 (see Eqs. (1) and Tab. S1) are uniformly distributed in the logarithmic space around the original parameter values 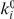 where the variability is controlled by the heterogeneity parameter 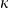: 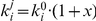 with 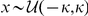, 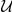 being a uniform distribution in the range 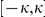. All individual parameters are randomly chosen without any intra- or intercellular correlations. Examples of individual oscillators are shown in figure 3A. We tested different values of 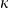 between 0.025 and 0.3 (Fig. 3B–C) and observed that small values of 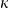 generate a population where about half of cells have limit-cycle oscillations (Fig. 3B), but the pseudo-periods (defined as the average duration between two peaks, see *Models*) have little variability (Fig. 3C). On the other hand, large values of 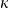 lead to high heterogeneity where some cells are overdamped and the pseudo-periods are broadly distributed. In the intermediate regime (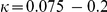), the results are not very sensitive to 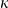, therefore for the different simulations, we chose a value of 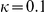. For this value, about 35–40% of the cells oscillate in isolation as observed experimentally [6] (Fig. 3D). The distribution of the pseudo-period of oscillation is centered on a value of 21.2 hours with a standard deviation of 0.7 hour (Fig. 3E) which is in the range of experimental results [35].

### The SCN as a network of oscillators

To form the SCN network, we supposed that the cells are connected with directed (unidirectional) edges through the dendrites. The upstream cell produces a neurotransmitter 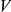 acting on a signaling cascade in the downstream cell that increases 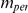 expression, the 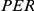 coding mRNA (Fig. 2). The effect of the incoming signals from the different cells sums up until saturation (see Eq. (3) in *Models*). The coupling parameter 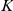, that represents the strength of the effect of 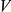 on PKA activation in the downstream cell, was set to a value of 0.5 for most simulations (the effect of the value of 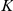 will be discussed later). Note that, although 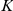 is identical for all cells, intercellular heterogeneity causes variability in the connection strengths due to differences in the dynamics of the species involved in the cell-cell communication (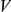, PKA and CREB).

**Figure 4:**
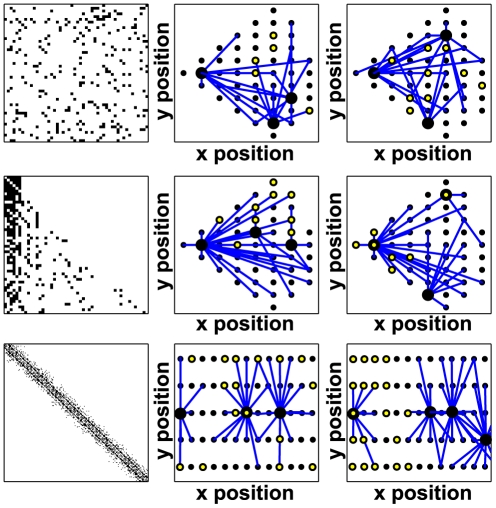
The different topologies tested. Three types of networks are used in this work: random architecture (first row, 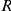), scale-free architecture (second row, 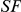) and local connections (last row, 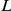). Note that the spatial distribution plays a role only in the ‘local’ networks. In the first column the corresponding adjacency matrix 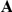 is shown (a black square at position 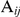 represents a connection from the 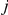-th to the 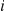-th cell). In the second column, a representative network is drawn showing outgoing edges (blue lines) from certain cells (larger black circles) and a random distribution of light-sensitive cells (small yellow dots in the black circles). These networks are named 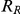, 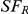 and 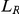 respectively. In the third column, the network has a biased distribution of light-sensitive cells, either on the cells with a higher outgoing degree (for random and scale-free networks, first two row, named respectively 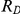 and 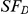), or spatially localized (for local networks, last row, named 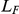).

In this analysis, we mainly focused on the effect of the network topology on the synchronization properties and ignore the effect of individual parameters. We selected three generic types of networks: random connections between cells (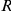), scale-free distribution of the outgoing edges (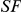), or local connections only (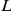) (see *Models* and Fig. 4). Each type of network contains 200 cells and we tested different values of 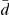, the average number of edges per cell, ranging from 3 to 15. For simulations with light, in agreement with the experimental observations [13], we assumed that only 20% of cells, on average, are light-sensitive and the distribution of light-sensitive cells can be either random (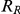, 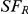 or 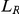 respectively, second column in Fig. 4), biased to favor the cells with the highest outgoing degree (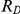 or 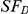) or spatially localized in the case of the local topology (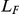, third column in Fig. 4). In the six topologies, the average degree and the fraction of light-sensitive cells are identical to allow a fair comparison (see Tab. S2).

**Figure 5:**
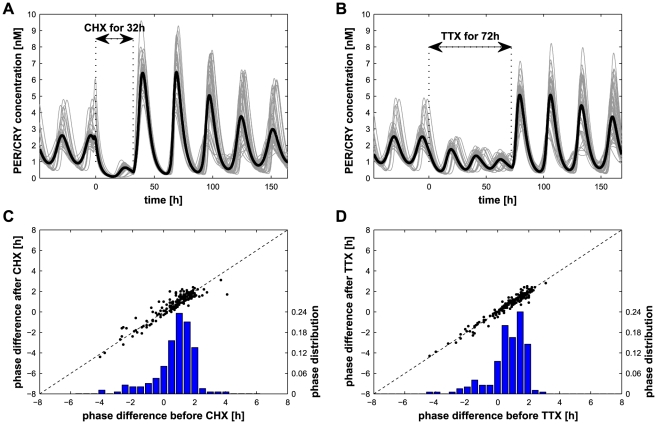
Effect of CHX and TTX treatments. (A) Resynchronization in constant dark for a scale-free network with 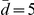 and 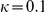 after addition of CHX (no protein production) for 32 hours and its removal. (B) Same network exposed to TTX (no cell-cell communication) for 72 hours. Gray lines represent PER/CRY concentration in individual cells and the thick black line is the population average 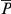. (C–D) Comparison of the phases of the individual oscillators before (x-axis) and after (left y-axis) CHX (C) or TTX (D) exposure (black dots). In blue, the distribution of the phase prior to the perturbation is plotted on the right y-axis. A positive phase difference corresponds to a phase advance of the individual cell compared to the average 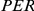 concentration.

We first performed the following *in silico* experiments [9], [11], [36]: interruption of the protein production due to an administration of cycloheximide (CHX) or interruption of the cell-cell communication through exposure to tetrodotoxin (TTX, see *Models* for implementation), both in a network without light entrainment. Our results are consistent with the experiments: cells stop oscillating upon exposure but quickly resynchronize after CHX (Fig. 5A) or TTX wash-out (Fig. 5B). The phase of the individual oscillators (measured at the stationary state, about 30 cycles after the perturbation) is conserved after both CHX (Pearson's 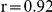, 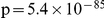 in Fig. 5C) [11] and TTX (Pearson's 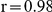, 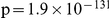 in Fig. 5D) perturbations [36]. Results in figure 5 were made using a scale-free network, but the other topologies tested in this work (random and local) display similar results. As previously reported [37], the period of the individual oscillators is negatively correlated with the difference between the phase of the same oscillator and the phase of the network (Pearson's 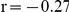, 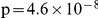, Fig. S1).

### Synchronization properties of the different network topologies

**Figure 6:**
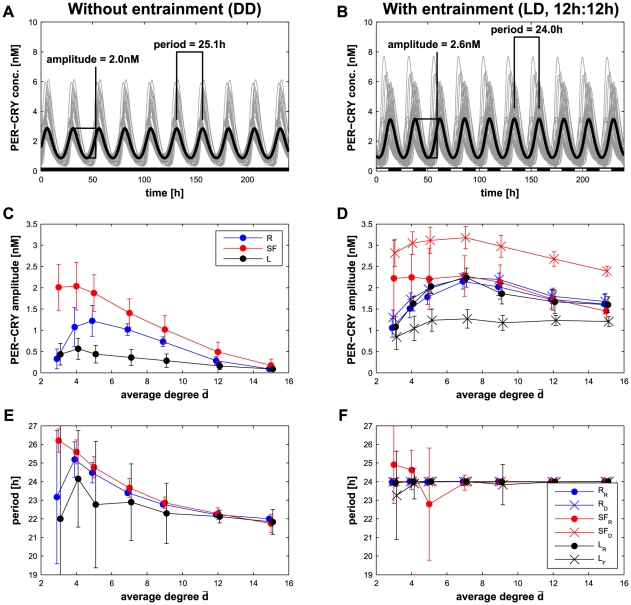
Properties of the synchronized network. (A) Synchronization in constant darkness (DD) of a scale-free architecture 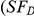) network with 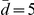 and 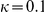. The measured properties are the amplitude of the 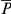 oscillations and the period of these oscillations. Each gray line represents the concentration of 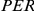 in an individual cell; the thick black line is 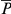. (B) Synchronization in 12 h∶12 h light/dark conditions (LD) for the same network as (A). (C–D) Amplitude of the 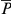 oscillations in the DD (C) and LD (D) conditions for different network types as a function of 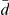. (E–F) Period of the 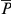 oscillations in the DD conditions (E) and in LD conditions (F) for different network types as a function of 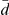. The amplitude and period in the LD conditions for the 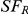 and 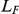 networks (D and F) shows large variability because some networks with low connectivity are not properly entrained. This weak entrainment (due to the architecture) induces amplitude modulation and biases the results. In C–F, error bars represent the standard deviation for the results of 30 different networks of the same type. The network types are abbreviated as 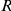 for random, 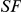 for scale-free, and 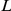 for local; the subscript 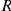 stands for a random distribution of the light-sensitive cells and the subscripts 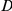 or 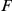 for a biased distribution as shown in figure 4.

**Figure 7:**
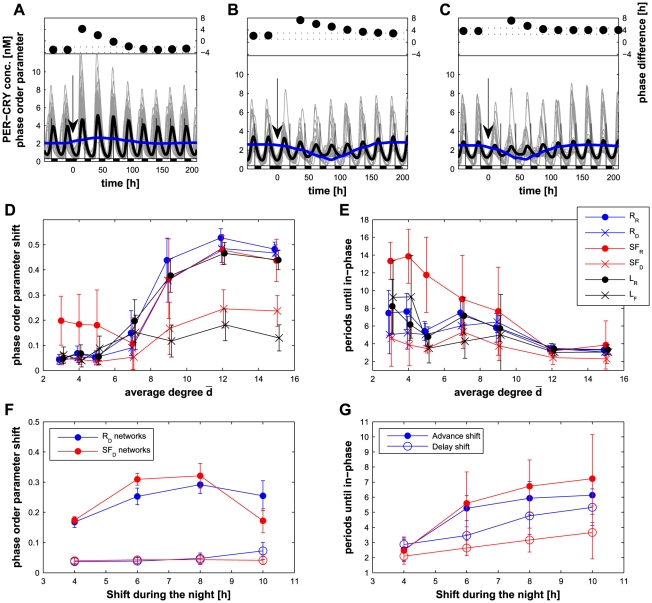
Effect of a jet lag on the SCN model. (A) In the case of an 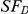 network with 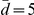, the 8-hour shift due to a long night (at 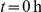) affects the phase of the peak of 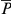 (black line) for about 3 cycles. Before the jet lag, the peak occurs about 4 hours after the night. In the first 3 cycles, the peak is in the late day and regains its initial phase at the fourth cycle (top inset, a positive value implies a phase advance). Throughout this perturbation, the cells remain well synchronized: the phase order parameter (blue line) is even increased. (B) For an 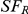 network with 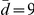, the system needs about 6 cycles to recover its correct phase and suffers a strong desynchronization. (C) For an 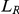 network with 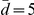, the system needs only 4 cycles to recover the phase, but cells are strongly desynchronized and the amplitude of oscillations decreases significantly. (D–E) Decrease in the phase order parameter after the jet lag (D), and number of cycles needed for the phase to be within 1 hour of the phase prior to the jet lag (E) as a function of the network type and the average degree. In both plots, lower values correspond to a faster adaptation: 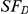 networks show better results for both properties. Note that the results for the 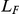 networks are less relevant as the oscillation amplitude is low (Fig. 4D). Results using other types of jet lags are plotted in figure S5. (F–G) Decrease in the phase order parameter (F), and number of cycles needed for phase resynchronization (G) after the jet lag as a function of the shift in hours for 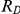 and 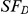 networks with 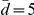. In all cases, except 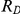 with 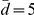, advance shifts (dots) have a stronger impact than the corresponding delay shifts (circles).

To compare the different networks, we focused on the concentration of the PER/CRY complex (variable 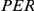) averaged over all cells (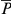, see Eq. (7) in *Models*) and evaluated its amplitude and period of oscillation (see Fig. 6A and *Models*). As the networks are randomly generated, all results in figures 6 and 7 represent the mean of the value measured over 30 different networks. We also defined two different order parameters as described in the *Models* section, equations (8) and (9): the state order parameter 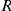 that measures how synchronized are the individual oscillators over the length of the simulation, and the phase order parameter 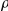, that measures how the individual oscillators are in phase at a given time (note that this measure is independent of the magnitude of the amplitude). It is worth noting that the coupling function implies that a cell always acts on the dynamics of its downstream cells even if they are synchronized. This differs from a diffusive interaction (e.g. Kuramoto oscillators [28], [38] in which the coupling depends on the phase difference) for which synchronized oscillators have no influence on each other. This property, along with cellular heterogeneity, prevent the application of theoretical results found in the literature [39], [40], and require a numerical analysis.

In the following, we distinguish two different conditions of simulation: in constant dark (DD, no entrainment, Fig. 6A) or in a light-dark cycle (LD, period of 24 hours with 12 hours of entrainment, Fig. 6B). The results of the mean amplitude and the oscillatory period for different network architectures (average of 30 randomly chosen networks for each condition) are shown in figure 6C–F. Considering the random networks, the amplitude of 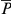 oscillations strongly depends on the average degree 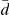 with the maximum value seen for an intermediate connectivity: 5 edges per cell for the case without entrainment (Fig. 6C) and 7 with entrainment (Fig. 6D). This dependence on the number of edges is also reflected in the order parameters 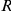 and 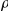 whose values are maximal for an intermediate connectivity of 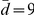 (Fig. S2). The period in DD conditions is around 25 hours for low connectivity which is closer to experimental evidence [35] than the period of 22 hours found in highly connected networks. In LD conditions, all random networks have a 24-hour period, reflecting proper entrainment by light.

For the scale-free networks, the amplitude the system exhibits in darkness is the largest of all topologies, also for very low connectivity (Fig. 6C). It drops when increasing the number of edges and converges to the results of the other networks. The period of 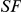 networks without entrainment is around 26 hours for 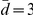 and decreases down to 22 hours for higher connectivity as for the random networks. In light-entrained conditions, a significant difference can be noticed between the case where the light-sensitive cells are randomly distributed (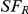), and the case where the cells with high outgoing degree are light-sensitive (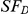). Although both network types show large amplitude, the 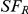 networks do not systematically have the same period as the entrainment signal for 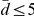 because a significant fractions of the cells are not located downstream of a light-sensitive cell. On the other hand, 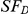 networks have a period of 24 hours for all tested 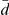 values which means that 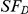 networks are more suitable to represent the SCN.

For the local topology, we observed that, without entrainment (left column in Fig. 6), local networks have a low amplitude due to a lack of synchronization throughout the network (see also Fig. S2A–B). Clearly, since the connections are only local (Fig. 4), the network does not have the small-world property [25]. On the other hand, with light entrainment, local networks with a random distribution (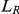) of the light-sensitive cells have ample oscillations and a 24-hour period. In this specific case, due to the random distribution of light-sensitive cells, most of the cells are directly downstream of a cell entrained by light even for small 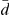 (Fig. S3). In the case where the light-sensitive cells are closely localized (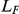), the entrainment efficiency is weak and the oscillation amplitude of 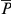 is low.

These results suggest that, in constant dark, the scale-free, and to a lesser extent, the random architectures with an intermediate connectivity (5–7 edges per cell on average) seem to represent the experimental data best. In contrast, local architectures as defined in our work impede an efficient synchronization of the cells and therefore show small oscillations. In LD conditions, the distribution of the light-sensitive cells plays a significant role and the networks that have a smaller average distance to a light-sensitive cell (Fig. S3), i.e. the 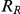, 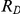, 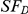 or 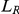 networks, show a larger oscillatory amplitude (Pearson's 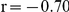, 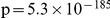 over all networks types and average degrees).

### Effect of the coupling constant on the synchronization properties

The relationship between the average number of degrees and the amplitude in both DD and LD conditions (Fig. 6C–D) suggests that a strong connectivity is detrimental for system performance. This raises the question of how the value of the coupling constant 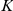 affects the network oscillations. While maintaining 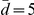, a stronger coupling constant (larger 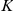) decreases the amplitude and the period of oscillations in DD conditions (Fig. S4A, C). In light/dark conditions, the relation between 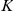 and 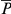 oscillation amplitude follows a bell-shaped curve, the maximum of which depends on the network type. For 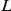 networks, a weak coupling (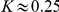) is optimal, whereas an intermediate coupling (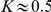) favors 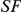 networks and a strong coupling (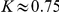) is preferred for random networks. Note that, for most of the 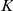 values the performance ranking of the network types remains the same (scale-free networks showing largest amplitude). In addition, although 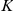 can be fine-tuned to increase the performance of a given network type, the results we obtained with 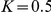 are qualitatively similar to results with other 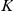 values which is why 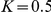 will be used for further analyses.

### Resynchronization and adaptation of the network after a jet lag

We then considered the case of a perturbation in the entrainment pattern of light/dark alternation. Since one of the goals of the circadian clock is to ensure the adaptation to the day-night cycle, an efficient clock should resynchronize rapidly after a jet lag. We chose the case of an 8-hour shift resulting in a long night of 20 hours, followed by the regular 12 h∶12 h LD cycle. As a measure of resynchronization, we considered the number of cycles until the system recovers, i.e. has a phase difference between the peak of 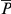 and the beginning of the night similar to the one prior to the jet lag [41]. We also determined the maximal decrease of the phase order parameter after the jet lag as a measure of how the individual cells desynchronize as a consequence of the jet lag.

As shown in figure 7A–C, the effect of a long night depends on the network type. In the case of an 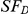 topology, the synchronization of the system is hardly perturbed (blue line in Fig. 7A) and the phase difference between the peak of 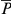 and the beginning of the night recovers its value prior to the jet lag in about 3 cycles. On the contrary, the 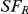 network needs about 6 cycles to regain the proper phase with a strong decrease of synchronization (Fig. 7B). For the 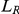 network, although the system experiences desynchronization, the phase difference is recovered in about 4 cycles (Fig. 7C). A systematic analysis of the different network types shows that random networks (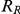 and 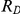) and scale-free networks with biased distribution of the light-sensitive cells (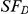) undergo very little desynchronization (Fig. 7D–E). Note that the results for the 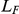 networks are less relevant because these networks display very low amplitude.

In order to generalize the measured advantage of the 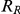, 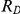 and 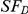 network types for resynchronization after a jet lag, we tested 3 other types of 8-hour shifts: a short night, a long and a short days. The results (summarized in Fig. S5) show that these three types of networks are also the best performers when experiencing other types of jet lags, but also that the long day or night (delay shifts) have less impact than the short day or night (advance shifts). We further investigated this difference between delays and advances for 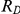 and 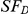 networks with 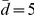. For different shifts ranging from 4 to 10 hours, long shifts induce longer resynchronization time (Fig. 7F–G), but additionally, the network resynchronizes significantly faster after a delay than an advance of the same shift duration (Wilcoxon's 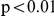 with n = 30 for all shifts and both networks, expect for 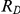 with a 4-hour shift). Remarkably, this corresponds to experimental evidence on mice [42] and physiological observations showing that recovery from a jet lag due to westbound flights (long day or night) is easier than recovery from eastbound ones [43].

### Coupling two network types to model the VL and DM regions of the SCN

**Figure 8:**
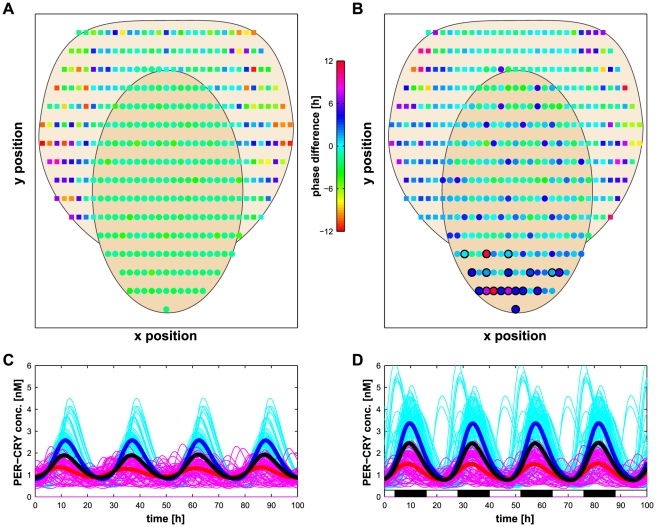
Simulation of the SCN composed of two regions in DD and LD conditions. Simulation of the SCN with different architectures for the VL and DM regions (an 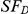 coupled to an 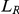 network, see Fig. S7 for a sketch of the topology), where the coupling constant 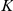 between the DM cells is 0.15 and the DM cells are oscillating faster (see *Models*). (A–B) Phase difference of the cells in DD (A) and LD (B) conditions. Dots represent cells of the VL (beige region), whereas DM cells are small squares (light yellow region). Phase difference is color encoded: green corresponds to a phase delay, blue to a phase advance and red to antiphase (see also Fig. S13). (C–D) Concentration of 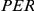 in the individual VL (cyan lines) and DM cells (magenta lines), as well as the average over the VL cells (thick blue line), the DM cells (bold red line), and over the entire SCN (thick black line) in DD (C) and LD (D) conditions.

The next question we addressed concerns the separation of the SCN in two different regions, namely ventro-lateral (VL) and dorso-medial (DM). Experimental observations have shown that the VL is entrained by light but oscillates with large amplitude even in dark conditions [11], [12]. These properties closely correspond to networks with 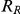, 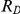 or 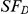 architectures. On the other hand, the current consensus for the DM, is an entrainment through the VL and not directly by light [11]. Additionally, when detached from the VL, the cells of the DM hardly oscillate and are not synchronized. When looking for these features in the network types studied above, a local network with random distribution of the entrained cells seems to best represent the DM. In terms of geometry, the VL forms a core surrounded by the DM which would lead to the hypothesis that connections between the VL and the DM regions occurs locally on the border between the two regions. A biased distribution of the light-sensitive cells in the VL is also plausible as the SCN is located above the optical chiasm (Fig. 1) and thus the cells located in the lower part of the VL could be more sensitive to the light clues. Such configuration would allow a compact organization of the SCN without long neuronal connections (Fig. 8A–B and S7).

To test this hypothesis, we performed simulations of a SCN composed of two regions with the following properties. The VL is modeled by an 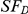 network composed of 200 cells and has an average value of 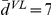 edges. Random networks are also able to produce ample oscillations in the VL (Fig. S6A, C), however the local networks are not plausible due to their low amplitude in DD conditions. For the DM, we chose a local network of 200 cells with 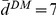 surrounding the VL region as other topologies would require long connections across the VL. Cells of the VL and the DM are heterogeneous with parameters distributed as previously (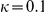). Entrainment of the DM by the VL is made by local connections with an average outgoing degree of 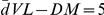 (Fig. S7). Note that this architecture implies that no DM cells can be upstream of a VL cell.

The simulations of this system show good synchronization and entrainment of the DM part in both dark and light/dark conditions (Fig. S8). However we saw a delay of the DM phase in comparison to the VL (Fig. S8B, D), which contradicts the experimental results [11]. To counter this problem, we used faster oscillating cells for the DM (see Eq. (6) in *Models*) as suggested by experimental data [44]. With this adjustment, the DM is not properly entrained by the VL because the free-running period of the whole DM is too short. This can be improved by decreasing 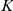 to 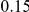 in the DM only (Fig. 8) which results in oscillations with larger amplitude (Fig. S4B) in LD conditions, as well as an increase of the free-running period (Fig. S4C). Additionally, reducing the coupling has also been suggested as a way of facilitating the entrainment [45]. With this configuration, the center of the DM is in phase with the VL and some exterior cells are in phase advance (Fig. 8A,B). When isolated from the VL, the DM cells are not synchronized (Fig. S9) which is in agreement with experimental observations [11]. Note that for a core formed of a random network, the DM is delayed in LD conditions despite these adjustments (Fig. S6B, D). This suggests that a scale-free architecture is the most plausible topology for the VL region of the SCN.

### Effects of perturbations on an SCN composed of two network types

**Figure 9:**
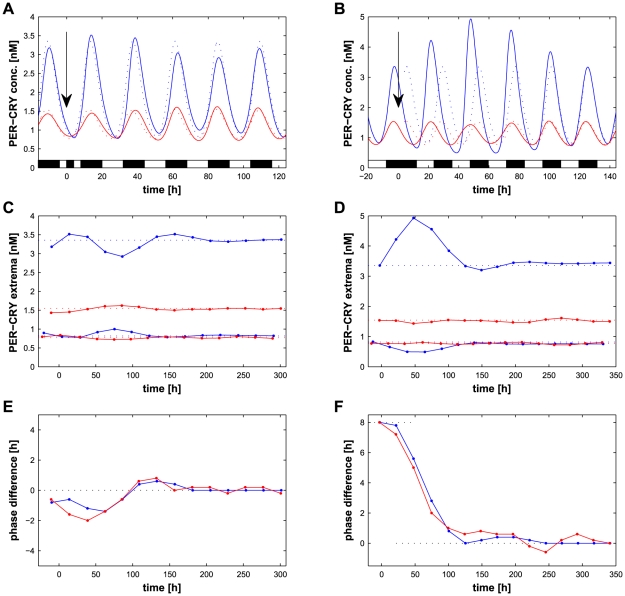
Effects of entrainment perturbations on the SCN composed of two regions. (A) Effect of a 4-hour interruption of the light entrainment during the day (see arrow a 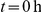) on VL (blue) and DM (red) average 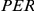 concentrations in the same SCN network as in figure 8. Dashed lines represent the unperturbed trajectories. (B) Effect of an 8-hour jet lag (long night, see arrow at 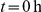) on VL (blue), DM (red) average 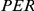 concentrations (same network). Dashed lines represent the unperturbed trajectory shifted from 8 hours. (C–D) Average concentration of 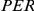 at the extremum (points) of oscillations after an interruption of the light entrainment of 4 hours during the day (C), corresponding to panel A, or after a jet lag of 8 hours equivalent to a long night (D), corresponding to panel B. The average of VL cells is in blue and of DM cells in red. Dashed lines show unperturbed values. (E–F) Phase shift of the peak of 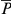 for the VL (blue) and DM (red) after an interruption of the light entrainment of 4 hours during the day (E), corresponding to panel A, or after a jet lag of 8 hours equivalent to a long night (F), corresponding to panel B. A positive value corresponds to a phase advance.

A possible advantage of a division of the SCN in two regions can be to filter disturbances of the entraining LD cycle. To test this hypothesis, we perturbed the light inputs in two different ways and measured the effect on 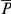 in the VL and the DM regions of the SCN. The first perturbation is an interruption of 4 hours of the light cue during the day (a pulse of light during the night has only a marginal effect and was therefore not studied further). In this case, (Fig. 9A), the amplitude of the average 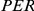 concentration over the VL cells rises before dropping by about 20%. The initial value is recovered after about 10 cycles (Fig. 9C). The phase is also affected, first delayed by about 1.5 hours and then advanced by the same value (Fig. 9E). However, the amplitude of the DM part is hardly affected by the perturbation, although the maximal phase shift is similar. To quantify the effect of the perturbation, we defined 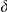 as the average normalized difference between the peaks and the stationary peaks over 300 hours after the perturbation (see Eq. (10) in *Models*). Averaged over 30 different networks, the effect of the 4 h light interruption on the VL is 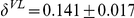, which is 33% more (Wilcoxon's 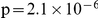) than on the DM: 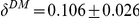.

The second perturbation studied is, as previously, a jet lag of 8 hours occurring during the night (resulting in a long night of 20 hours). The VL cell reacts strongly by increasing the peak value of oscillations by about 50% (Fig. 9B,D). As already measured (Fig. 7E), the phase of the VL adjusts precisely to the new entrainment pattern in about 4 cycles (Fig. 9F). The phase of the DM follows the VL within one cycle reaching the correct phase in 5 cycles. Here also, we observed a strong difference between the VL and the DM parts of the SCN: 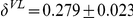 whereas 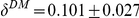 (Wilcoxon's 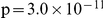). These results suggest that the separation of the SCN in two parts with different topologies allows the DM region to have a lower sensitivity to short entrainment perturbations while at the same time better adapts to long term changes than a network formed of a unique topology such as the VL.

## Discussion

In this work, we addressed the question of the organization of the neuronal cells in the SCN by assessing the synchronization properties of different types of networks. In these networks, each cell is a circadian oscillator but the population shows heterogeneity in its oscillatory behavior as observed experimentally [7], [6]. We found that, in general, the network is able to cope with cellular heterogeneity and the system oscillates with large amplitude and a period slightly longer than the individual period which is consistent with *in vitro* measures [46].

Our results show that the architecture of the network, independently of the number of cells in the network (Fig. S10), plays a significant role in the synchronization properties. In general, we observed that a strong connectivity, either due to a high number of connections or a strong value of the coupling constant 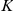, is detrimental for the amplitude of oscillations. The distribution of the edges also plays a critical role: Vasalou *et al.* [25] already observed that small-world networks are better synchronized than networks with local connections. Our results not only confirm that random networks better synchronize than our local networks, but also show that scale-free networks exhibit larger oscillations and better synchrony with fewer connections in DD conditions. In LD conditions, a strong correlation exists between the average distance to a light-sensitive cell and the performance of the network (Fig. S3). In our work, two types of networks result in a short average distance and therefore ample oscillations in LD conditions: (1) networks with a uniform degree distribution (local or random) and uniformly distributed light-sensitive cells, or (2) scale-free networks where the cells with high outgoing degree are light-sensitive.

These results were obtained with a variability 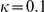 as the distribution of individual cells properties matched experimental data. We now briefly comment on the effect of the value 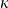 for the different types of networks. To simplify the analysis, we varied 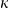 only for networks with an average degree of 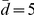 as all types of networks show good performance for this value. In both DD and LD conditions, although the synchronization increases, oscillation amplitude remains similar for values of 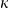 between 0 and 0.1 (Fig. S11A–B), reflecting that the networks can efficiently cope with some cell-to-cell variability and that a tight tuning of individual oscillators is not necessary. This property holds for all types of networks. Cell heterogeneity also induces phase fluctuation [47] and we found a rather weak correlation (Pearson correlation coefficient 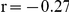) between individual phase differences and the period of the cellular oscillators (Fig. S1) which is closer to experimental observations [47] than the high correlation reported for simpler models where heterogeneity was only introduced at the level of the period [37].

One of the properties of the circadian clock is adaptation to changes in the entrainment pattern for example after a jet lag or a long period of dark (hibernation). Although circadian rhythms and chronotherapy play an important role in medicine, the specific case of jet lag has only been marginally discussed in the modeling literature [41]. Our contribution to this question shows that the network topologies are strongly related to the resetting of the SCN with an advantage for small-world networks (such as random 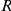 or scale-free networks with biased distribution of light-sensitive cells, 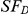) with an intermediate connectivity of 5–7 edges per cell. When comparing our results to experiments [42], [43], we observed that the 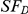 networks are closer to the experimental results where resynchronization is fast (2–3 cycles) for delay, and slower (4–5 cycles) for advance in the entrainment, confirming the observations that the circadian rhythm is more affected by eastbound than westbound-induced jet lags. It is also interesting to notice that a heterogeneous cell population seems to enhance resynchronization after a jet lag for the 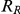, 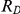 and 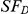 network types (Fig. S11C–D). Remarkably, experimental observations already suggested that the SCN regional heterogeneity and the multiple phase relationships among SCN cells could contribute to the photoperiodic adaptation [48]. Alternatively, a different entrainment pattern with a shorter light exposure (diurnal duration of 8 hours with a period of 24 hours), results in ampler oscillations than a system with a 16-hour light exposure especially for 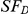 networks (Fig. S12), which is once again consistent with experimental observations [49].

Finally, the last and probably most ambitious part of this work consisted of coupling two networks with different properties to mimic the two regions of the SCN, namely the ventro-lateral and the dorso-medial parts. From our previous results, we selected a network combination that matched experimental facts: namely a core (VL) that is entrained by light and oscillates on its own, and a shell (DM) that can have sustained oscillations only while entrained by the VL. A scale-free network with biased distribution of the light-sensitive cells for the VL combined with a local network for the DM results in the desired properties with minimal connections. To more accurately match experimental data, we had to decrease the period of the cells in the DM as well as their coupling strength. With these adjustments, we obtained waves of 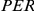 expression through the SCN (Fig. S13) as observed in cultured SCN slices [47], [50]. Other combinations of parameters can possibly reproduce the properties of the VL and DM parts but our exploration of the different types of networks was not exhaustive, due to high number of possible combinations. We nevertheless tried different types of connectivity for the DM as well as different distribution of the edges (allowing longer connections) and eventually obtained a valid model of the SCN that can be used for further analysis. In this work, we found that combining two networks with different connectivity properties (both in the topology, the strength of connections and the oscillation speed of the individual cells) showed better results than a homogeneous network. These results may provide insight on why different neurotransmitters are found in the different regions of the SCN.

Our results, proposing an optimal organization for the SCN, represent a step toward the understanding of the brain topology [51]. In practice, we can think that the neurons sensitive to light increase their number of connections to other cells in the SCN to form a scale-free network, an architecture already observed in *C. elegans* [52]. With such architecture, our model is able to reproduce many experimental results including the difference in recovery time between eastbound and westbound-induced jet lags, the larger amplitude for short days, and the distribution of the phase differences in the VL and DM regions of the SCN. The next stage in the SCN modeling would be to study how these topologies scale for a few thousands of cells in three dimensions [50]; indeed our hypotheses of a scale-free core with a surrounding shell should hold if the number of connections between the core and the shell remains sufficient.

Further studies could take into account additional sources of noise such as the molecular noise due to the low number of molecules involved in the generation of circadian oscillation in a single cell [53]. This approach could help to determine whether circadian oscillations at the level of a single cell are noisy self-sustained oscillators or damped oscillators driven by noise as current single cell bioluminescence data are not sufficient to discriminate between the two hypotheses [54]. Other sources of variability such as differences in the light sensitivity, or in the cellular coupling [55] along with correlations in the parameter variability can impair or, on the contrary, contribute to the sustainability of the circadian oscillations [56]. Indeed, heterogeneity in the periods has also been shown to help the population of globally coupled Goodwin-like oscillators to respond in a more coherent way to the external light-dark cycle [57]. Future work could also include more details on the molecular mechanism involved in the signaling pathway to explicitly study the consequence of a loss of cAMP circadian production [24]. Another direction would be to analyze the role of the network topology on the robustness of the oscillations with respect to noise as well as other perturbations like mutations [58]. Finally, other oscillator models [30], [32] should be tested and if our predictions (high connectivity is detrimental, the DM is less perturbed than the VL) are proven to be independent of the model, these results may have interesting medical applications and would be worth being studied experimentally in the context of circadian disturbances.

## Models

### Cellular oscillator

Using the generic parameters 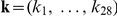, the equations of the Becker-Weimann model [23], [24], extended to account for the receptor signaling cascade are (1). Note that, in the network, each parameter of the cell 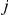 has a specific values 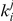 randomly drawn as described in equation (5) and Table S1.
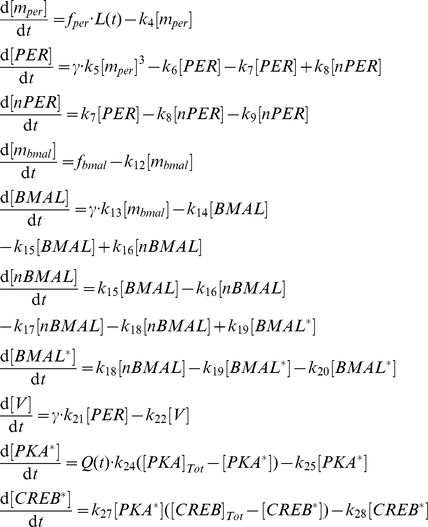
(1)Note that no distinction between PER and CRY is made. Thus, 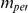 denotes both Per mRNA and Cry mRNA, 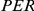 the PER/CRY cytosolic protein complex, and 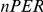 the PER/CRY nuclear protein complex.

The regulated transcription rates of the Per/Cry and Bmal1 genes are modeled by the phenomenological functions 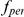 and 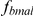, respectively:
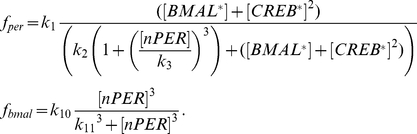
(2)


Parameter 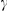 allows us to modulate the protein production rate. By default, 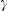 is kept equal to 1. In presence of cycloheximide (CHX, a toxin used experimentally to decrease protein production), 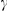 is decreased to 0.01.

The effect of light is expressed by the function 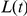 which is a multiplicative smoothed square wave that oscillates between 1 and 2, scaled by 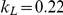 [23], [59], with a period 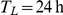 simulating a 12 h∶12 h Light/Dark cycle:
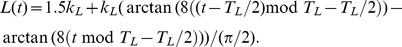
For parsimony, we assumed that the neurotransmitter 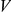 is produced in a linear manner by the PER/CRY complex, but more complex rate functions such as Hill terms can produce equivalent results.

### Intercellular coupling

The effect of cell-cell communication 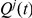 on the concentration of 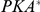 in the 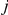-th cell (see Eq. (1)) depends on the sum of the neurotransmitters 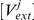 from the upstream cells:
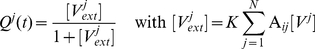
(3)with 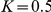, or 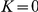 when tetrodotoxin (TTX, a neurotoxin that blocks cell-cell communication) is added to the medium. 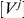 is the concentration of 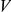 in the 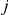-th cell, 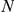 is the total number of cells, and 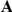 is the adjacency matrix of the network. As self-loops induce strong self-sustained oscillations in individual cell, a behavior that contradicts our hypothesis about cells in isolation, we deliberately prevented self-loops (i.e. 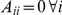). The topological characteristics of each set of 30 networks used for figure 5 are reported in Table S2. The simulations of the system are made with an ordinary differential equation integrator in MATLAB.

#### Random topology

Each possible directed connection 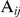, with 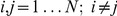 has a probability 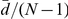 to exist. It results in an average degree of 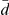.

#### Scale-free topology

The Barabási-Albert algorithm [26] is used to construct the scale-free network. We adapted it such that the outgoing distribution is biased but the incoming one is uniform. As in the Barabási-Albert algorithm, the construction of the network starts with a small nucleus of cells and then, during the ‘growth’ of the network, each new cell has 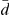 incoming connections whose upstream cells are chosen with a probability depending on their number of outgoing connections. Scale-free networks constructed in this manner possess the small-world property [26].

#### Local topology

In the local topology, cells are placed on a two-dimensional rectangular grid (each cell siting at an integer position) and the probability of forming an edge for cell 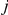 toward cell 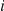 is a function of the Euclidian distance 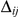 between the two cells 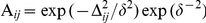 where 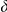 is adjusted to obtain an average of 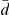 edges, i.e. 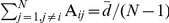.

### Parameter sets

Parameters of the oscillator model are adjusted to obtain damped oscillations with an individual period around 21 hours and sustained oscillations when entrained by light or stimulated by another cell through intercellular communication. Although there is no direct biological evidence for the values of each individual parameter, these values are in their biological range and the model show results consistent with experimental evidence for individual cell behavior [24], synchronization and entrainment. If we define the original parameter (see description in Tab. S1) as
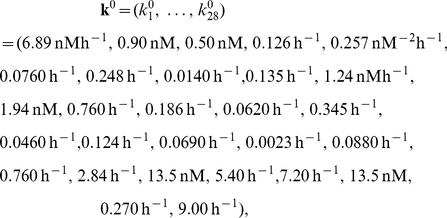
(4)with 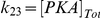 and 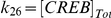, the parameters of the 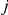-th cell are defined as:
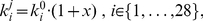
(5)where 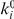 is the value in (4), and 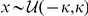 is a uniformly distributed random number in the interval 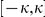. The variability parameter 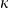 represents the amplitude of the rescaling of the parameters in the model.

For the model of the SCN composed of the VL and the DM regions, the cells in the DM are oscillating faster due to a rescaling of the kinetic constants in 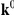 by a factor 1.15 prior to the draw of their parameters, i.e.
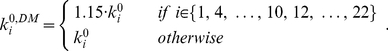
(6)


### Oscillator classification

To calibrate 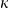 (Fig. 3), the oscillatory behavior of the individual cells should be classified even for damped oscillators. To this purpose, we defined the ‘pseudo-cycle’ as the trajectory between two peaks of 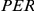 concentration. We considered that the cell stops oscillating (are damped) if the relative amplitude (amplitude of a pseudo-cycle divided by the maximal value) is lower than 0.1. With this statement, we defined the ‘pseudo-period’ as the average duration of the pseudo-cycles until the cell is damped. Cells are called ‘Not Oscillating’ if they are overdamped (i.e. no 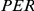 peak) after the entrainment is released.

### Measures of synchronization

A potentially important phenotype of the SCN is the average signal of the network. We considered the output to be the cell-average concentration of 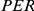, as experimental studies usually measure the luminescence of a reporter linked to the PER gene [50], [60]:
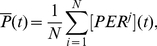
(7)and we measured the mean amplitude and the mean period (Fig. 6A) over a time of 100 hours (300 hours for the VL-DM model) after a relaxation time of 720 hours.

We also defined two order parameters to quantify the synchronization of the cells in the network. The first one is the state order parameter 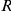 based on 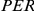 defined as [23], [61]:
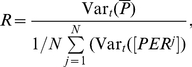
(8)where Var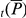 is the variance over time of 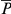.

However informativeness-wise, this measure is not always appropriate as cells have different individual amplitude due to parameter variability. Moreover it is based on an average over time which implies that it cannot measure how cells are synchronized at a given time point. We therefore defined another order parameter based on the phase of the individual oscillators. If 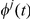 is the phase at time 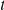 of the 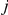-th cell evaluated with the Hilbert transform (see supplementary information of [62]) and 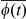 is the phase of the cell-average 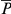, the phase order parameter 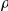 at each time point 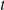 is:
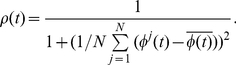
(9)


### Measure for the perturbation of the VL and DM parts

For the results of the model composed of two regions, we measured how the extrema differ from their stationary values. In order to account for the stationary amplitude, we normalized the difference. If the ensemble 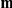 of local minima for 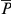 in the time interval 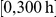 is:
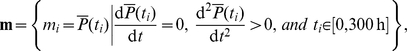
the ensemble 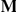 of local maxima:
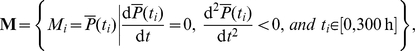
the cardinality of each ensemble 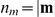 and 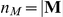, and the absolute minimum and resp. maximum before the perturbation:
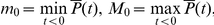
the value 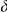 calculates as:
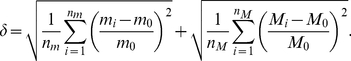
(10)This value is evaluated for the VL and the DM independently.

## Supporting Information

Figure S1
**Relation between the phase and the individual period.** (A–B) Phase difference between the peak of 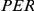 in the individual cells and the peak of the average 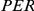 concentration corresponding to the main text figures 5A,C for (A) and 5B,D for (B). Oscillators with longer individual period show a delay in their phase represented by a negative value of the difference (Pearson's 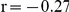, 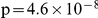). In this plot, the phase difference prior to the CHX or TTX perturbation is chosen but only marginal changes are observed when plotting the phase difference after the perturbation.(PDF)

Figure S2
**Order parameter for constant dark (DD) and 12 h∶12 h light/dark (LD) conditions.** (A–B) State order parameter in the DD (A) and LD (B) conditions for different network types as a function of 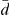. Error bars represent the standard deviation for the results of 30 different networks of the same type. (C–D) Phase order parameter in the DD (C) and LD (D) conditions for different network types as a function of 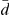.(PDF)

Figure S3
**Correlation between the average minimal distance to a light-sensitive cell and the amplitude of average** 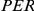 **concentration in LD conditions.** Each color is a different network type and the size of the points reflects the value of 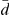 (ranging from 3 for the smallest points to 15 for the largest). Except for the specific network type 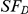 where the average minimal distance is around 0.9 for most networks with 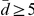, a negative correlation is observed.(PDF)

Figure S4
**Effect of the intercellular coupling parameter** 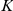 **on the network properties.** (A–B) Amplitude of the oscillations of 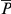 in the DD (A) and LD (B) conditions for different network types with 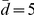 and 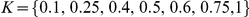. In DD conditions, the maximal amplitude is obtained with values around 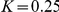 whereas in the entrained case (LD) 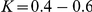 is optimal. (C–D) Period of the oscillations of 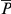 in the DD (C) and LD (D) conditions for different network types with 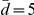 and same 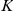 values as in A. In DD conditions, the free-running period decreases when 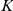 is increased whereas in LD conditions a larger 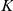 helps the 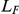 networks to be better entrained (other networks are already well-entrained).(PDF)

Figure S5 **Effect of different types of jet lag on the SCN model.** Decrease in the phase order parameter after the jet lag plotted against the number of cycles needed for the phase to be within 1 hour of the phase prior to jet lag. All subplots represent 8-hour shifts that induce either a short night (A), a long night (B), a short day (C) or a long day (D). Interestingly, shifts that correspond to a westbound flight (long night or day) have a smaller effects on the network than shifts corresponding to an eastbound flight (A,C).
(PDF)

Figure S6
**Simulation of the SCN with different architectures of the VL and DM regions (an** 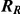 **network coupled to an** 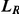 **one) with** 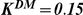 **and faster oscillating DM cells (see** ***Models*** **).** (A–B) Phase difference of the cells in DD (A) and LD (B) conditions. Dots represent the cells of the VL (beige region), squares DM cells (light yellow region). Green corresponds to a phase delay, blue to a phase advance and red to antiphase. (C–D) Concentration of 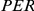 in the individual VL (cyan lines) and DM cells (magenta lines) and average over the VL (thick blue line) and DM cells (thick red line) as well as the entire SCN (thick black line) in DD (C) and LD (D) conditions.(PDF)

Figure S7
**Example of a network composed of the VL and the DM regions.** (A) Adjacency matrix where the top rows and left columns are for the VL cells and the bottom rows and right columns represent the DM cells (a black square at position 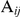 represents an connection from the 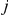-th cell to the 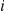-th one). (B) Cell positions and network architecture. Black dots represent the cells of the VL (beige region) and green squares the DM cells (light yellow region). Outgoing edges (blue lines) from certain cells (larger black circles) are also shown along with the light-sensitive cells (small yellow dots in the black circles).(PDF)

Figure S8
**Simulation of the SCN with different architectures of the VL and DM regions (an** 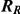 **network coupled to an** 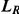 **one, see Fig. S7 for a sketch of the topology) with** 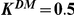 **.** (A–B) Phase difference between the cells in DD (A) and LD (B) conditions. Dots represent the cells of the VL (beige region) and squares the DM cells (light yellow region). Green corresponds to a phase delay, blue to a phase advance and red to antiphase. (C–D) Concentration of 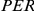 in the individual VL (cyan lines) and DM cells (magenta lines) and average over the VL (thick blue line) and DM cells (thick red line) and the entire SCN (thick black line) in DD (C) and LD (D) conditions.(PDF)

Figure S9
**Simulation of the cells of the DM with a coupling constant** 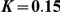 **isolated from the VL cells (corresponding to** **Fig. 8** **of the main text).**
(PDF)

Figure S10
**Effect of the number of cells on the network properties.** (A–B) Amplitude of the oscillations of 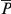 in the DD (C) and LD (D) conditions for different network types with 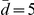 and a network size from 100 to 400 cells. (C–D) Period of the oscillations of 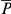 in the DD (C) and LD (D) conditions for different network types with 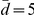 and a network size from 100 to 400 cells. Both properties are independent of the network size (p-value for correlation with the network size is above 0.05 for all combinations except for the amplitude of the 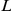 networks in DD).(PDF)

Figure S11
**Effect of the cellular heterogeneity parameterized by** 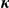 **on the network properties.** (A–B) Amplitude of the oscillations of 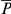 in the DD (C) and LD (D) conditions for different network types with 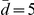 and 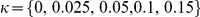. In the range 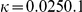 the amplitude is almost constant. (C–D) Period of 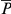 oscillations in the DD (C) and LD (D) conditions for different network types with 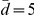 and 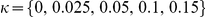. Periods of the 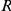 and 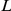 networks are hardly influenced by 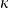, whereas 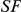 networks have a period closer to 24 hours for large 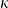 values. (E–F) Decrease in the phase order parameter after the jet lag (E) and number of cycles needed for the phase to be within 1 hour of the phase prior to the jet lag (F) for different network types with 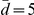 and 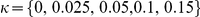. In general, cellular heterogeneity speeds up resynchronization of the network after the perturbation.(PDF)

Figure S12 **Changes in the oscillation amplitude for short and long days.** Amplitude in LD conditions for the six types of networks with either 8 hours (left bar) or 16 hours (right bar) of light (the period of the cycle remains 24 hours). All topologies have ampler oscillations for shorter days, consistent with [49].
(PDF)

Figure S13
**Wave propagation in the combined VL and DM model of the SCN.** (A) Normalized 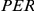 concentration in DD conditions as a function of time (y-axis) for the 400 cells (x-axis), corresponding to the network in figures 8 and S7. The cells closest to the optical chiasm (corresponding to the VL) are shown in the middle and the cells furthest (DM cells) are at the edge. (B) Normalized 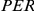 concentration in LD conditions for the same network.(PDF)

Table S1 **Value and description of the parameters of the model (adapted from** [**24**] **).**
(PDF)

Table S2 **Statistics of the networks used for results of** **figure 6** **.** All values are the average over 30 different networks. For the average degree, the value in parenthesis is the desired value.
(PDF)
