# Supplementary figures and images for: Effect of Network Architecture on Synchronization and Entrainment Properties of the Circadian Oscillations in the Suprachiasmatic Nucleus
Source: PLoS Comput Biol. 2012 Mar 8;8(3):e1002419. doi: 10.1371/journal.pcbi.1002419 (PMC3297560; doi:10.1371/journal.pcbi.1002419)

**A**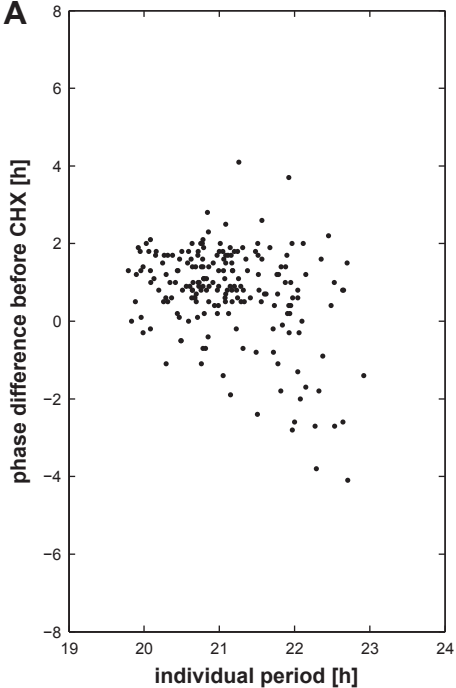**B**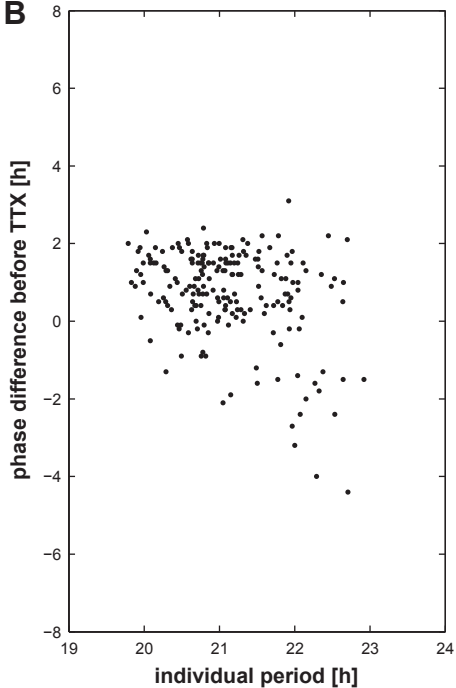

Supplement: Figure S1 — Relation between the phase and the individual period. (A–B) Phase difference between the peak of in the individual cells and the peak of the average concentration corresponding to the main text figures 5A,C for (A) and 5B,D for (B). Oscillators with longer individual period show a delay in their phase represented by a negative value of the difference (Pearson's , ). In this plot, the phase difference prior to the CHX or TTX perturbation is chosen but only marginal changes are observed when plotting the phase difference after the perturbation. (PDF) [file pcbi.1002419.s001.pdf]

**Without entrainment (DD)**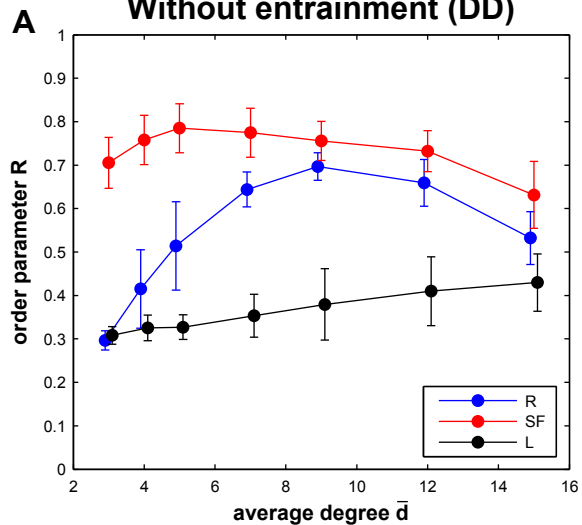**With entrainment (LD, 12h:12h)**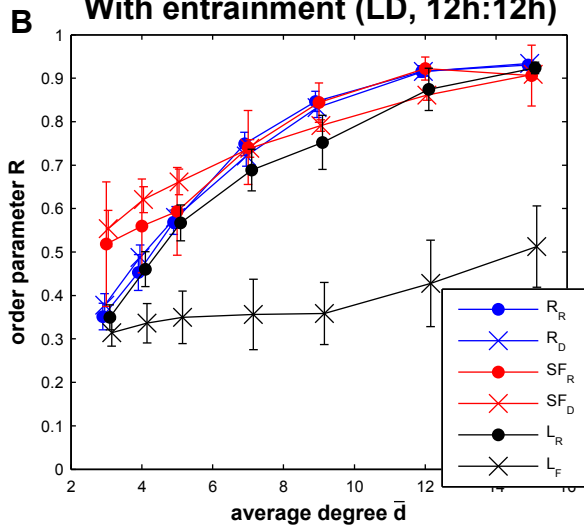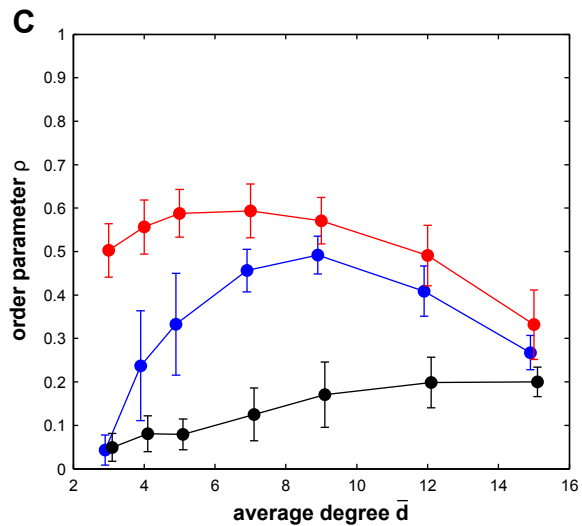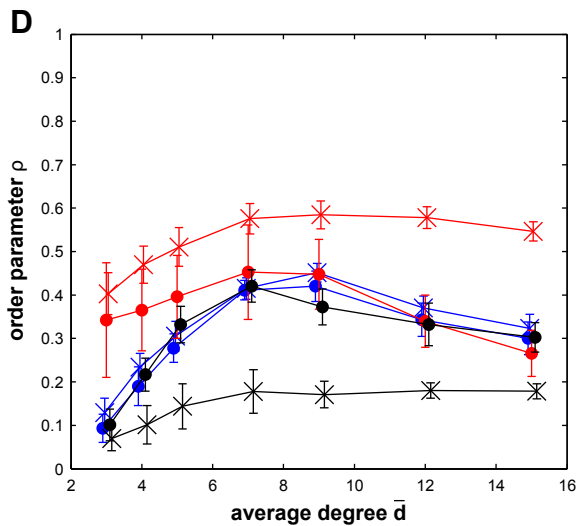

Supplement: Figure S2 — Order parameter for constant dark (DD) and 12 h∶12 h light/dark (LD) conditions. (A–B) State order parameter in the DD (A) and LD (B) conditions for different network types as a function of . Error bars represent the standard deviation for the results of 30 different networks of the same type. (C–D) Phase order parameter in the DD (C) and LD (D) conditions for different network types as a function of . (PDF) [file pcbi.1002419.s002.pdf]

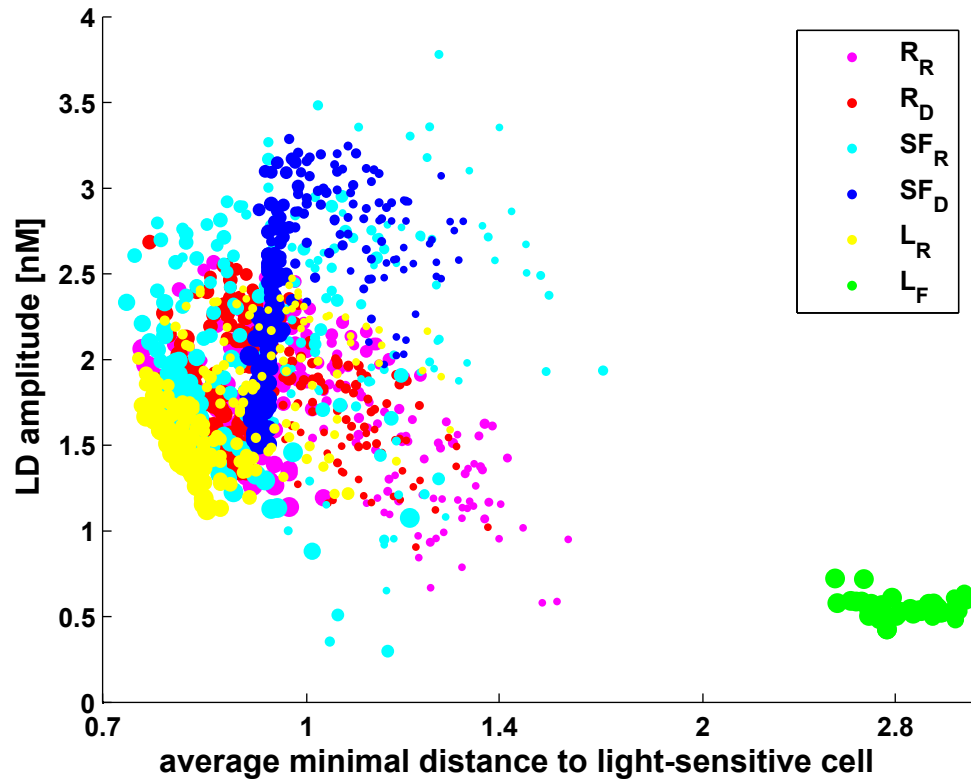

Supplement: Figure S3 — Correlation between the average minimal distance to a light-sensitive cell and the amplitude of average concentration in LD conditions. Each color is a different network type and the size of the points reflects the value of (ranging from 3 for the smallest points to 15 for the largest). Except for the specific network type where the average minimal distance is around 0.9 for most networks with , a negative correlation is observed. (PDF) [file pcbi.1002419.s003.pdf]

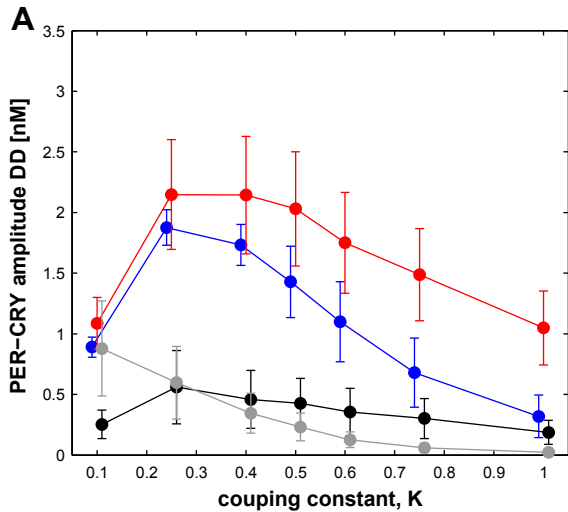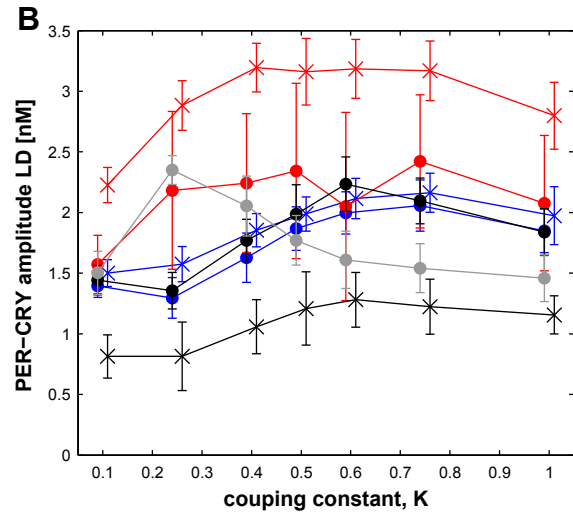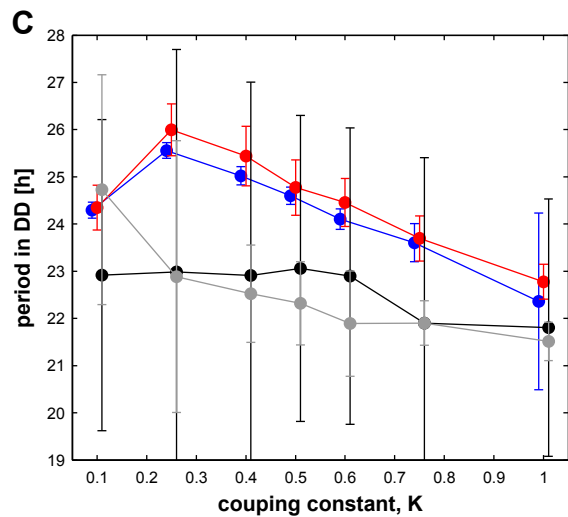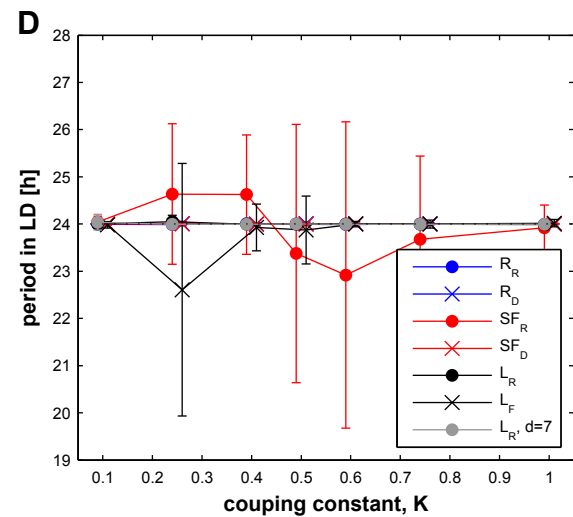

Supplement: Figure S4 — Effect of the intercellular coupling parameter on the network properties. (A–B) Amplitude of the oscillations of in the DD (A) and LD (B) conditions for different network types with and . In DD conditions, the maximal amplitude is obtained with values around whereas in the entrained case (LD) is optimal. (C–D) Period of the oscillations of in the DD (C) and LD (D) conditions for different network types with and same values as in A. In DD conditions, the free-running period decreases when is increased whereas in LD conditions a larger helps the networks to be better entrained (other networks are already well-entrained). (PDF) [file pcbi.1002419.s004.pdf]

Short night (4h)

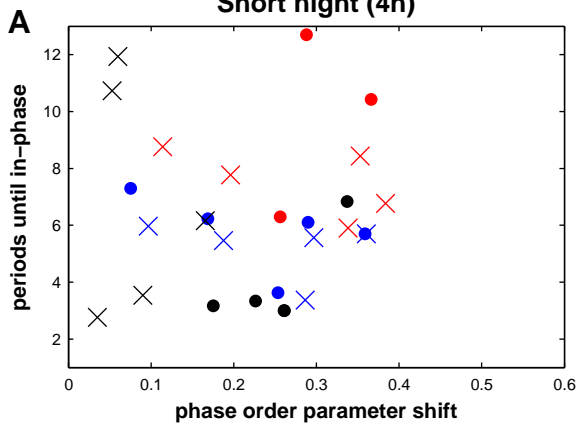

Long night (20h)

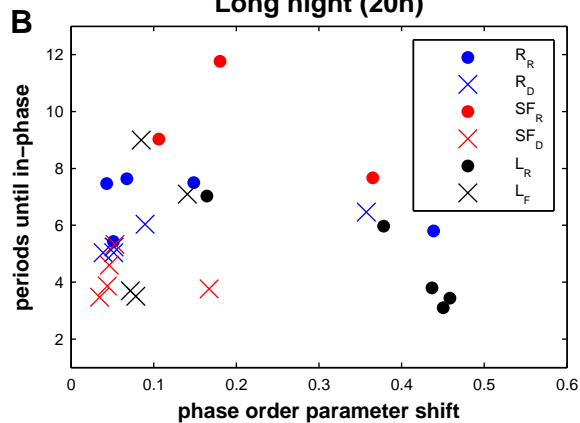

Short day (4h)

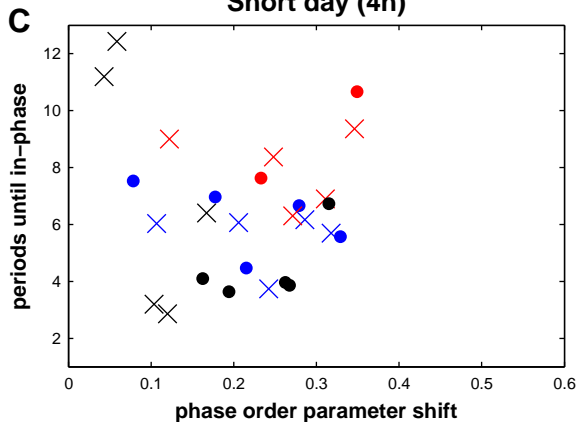

Long day (20h)

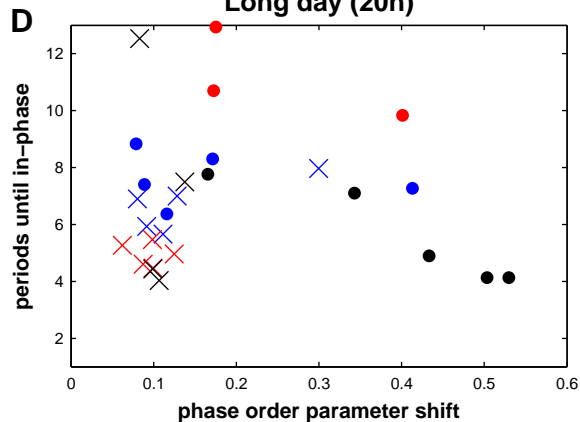

Supplement: Figure S5 — Effect of different types of jet lag on the SCN model. Decrease in the phase order parameter after the jet lag plotted against the number of cycles needed for the phase to be within 1 hour of the phase prior to jet lag. All subplots represent 8-hour shifts that induce either a short night (A), a long night (B), a short day (C) or a long day (D). Interestingly, shifts that correspond to a westbound flight (long night or day) have a smaller effects on the network than shifts corresponding to an eastbound flight (A,C). (PDF) [file pcbi.1002419.s005.pdf]

**A**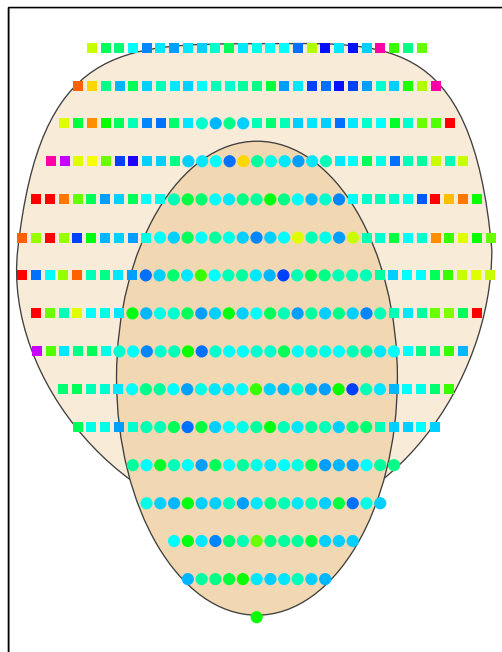**B**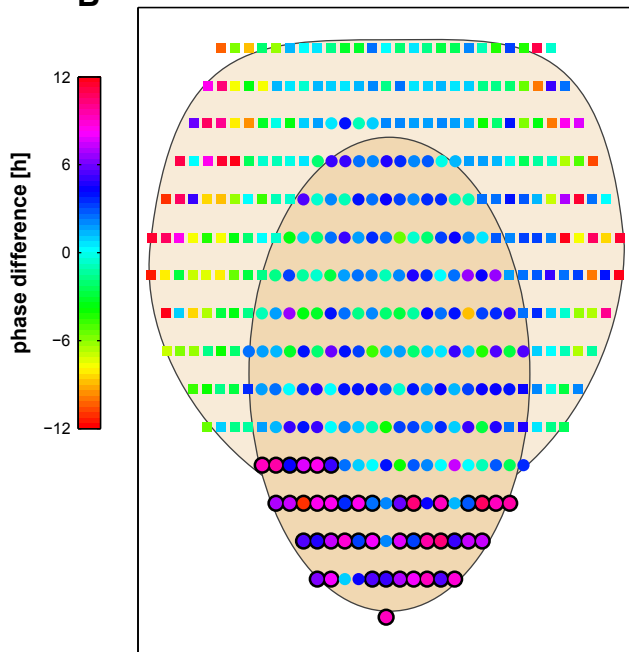**C**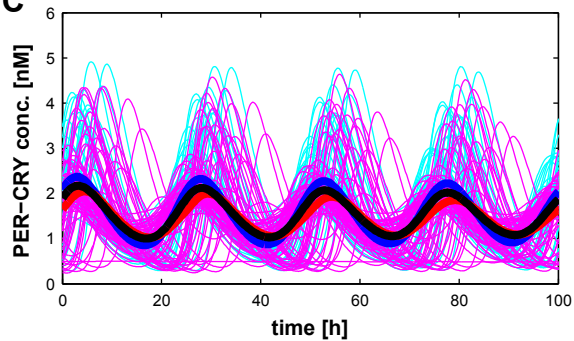**D**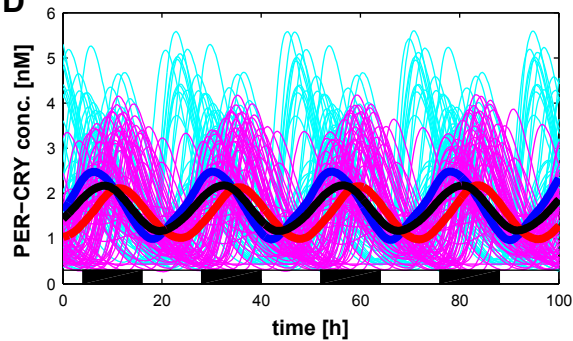

Supplement: Figure S6 — Simulation of the SCN with different architectures of the VL and DM regions (an network coupled to an one) with and faster oscillating DM cells (see Models ). (A–B) Phase difference of the cells in DD (A) and LD (B) conditions. Dots represent the cells of the VL (beige region), squares DM cells (light yellow region). Green corresponds to a phase delay, blue to a phase advance and red to antiphase. (C–D) Concentration of in the individual VL (cyan lines) and DM cells (magenta lines) and average over the VL (thick blue line) and DM cells (thick red line) as well as the entire SCN (thick black line) in DD (C) and LD (D) conditions. (PDF) [file pcbi.1002419.s006.pdf]

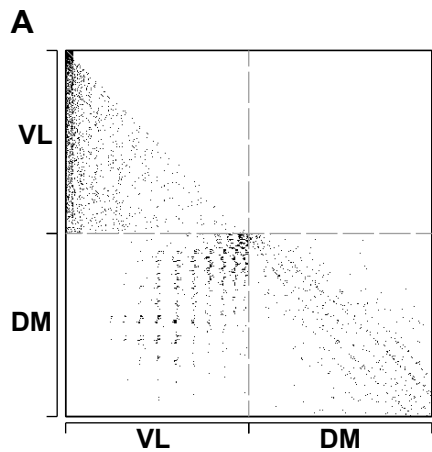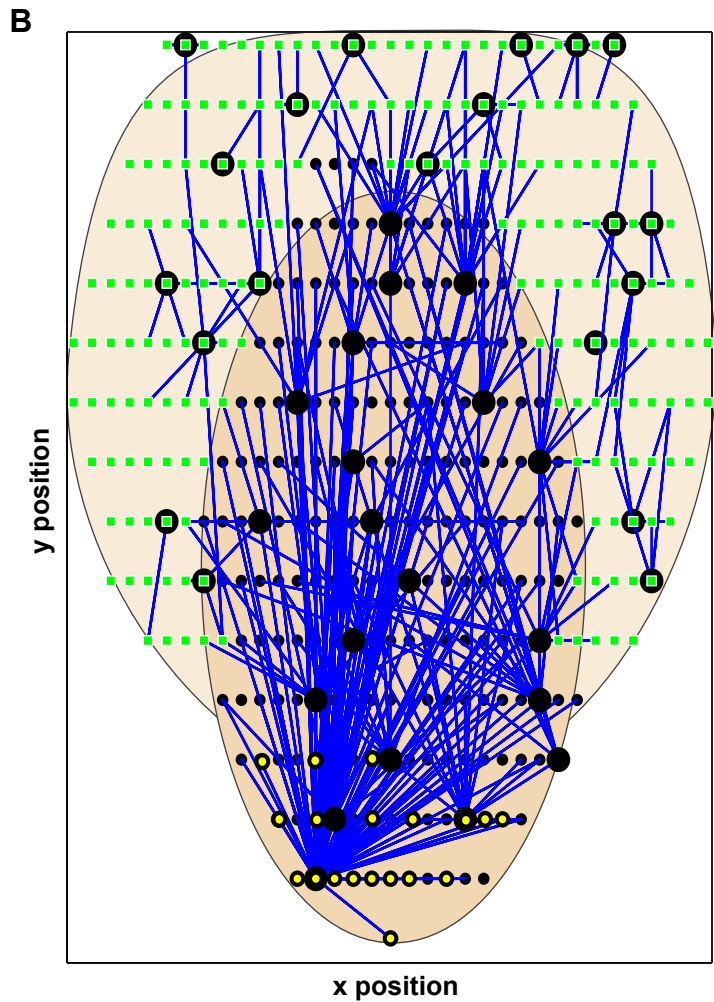

Supplement: Figure S7 — Example of a network composed of the VL and the DM regions. (A) Adjacency matrix where the top rows and left columns are for the VL cells and the bottom rows and right columns represent the DM cells (a black square at position represents an connection from the -th cell to the -th one). (B) Cell positions and network architecture. Black dots represent the cells of the VL (beige region) and green squares the DM cells (light yellow region). Outgoing edges (blue lines) from certain cells (larger black circles) are also shown along with the light-sensitive cells (small yellow dots in the black circles). (PDF) [file pcbi.1002419.s007.pdf]

**A**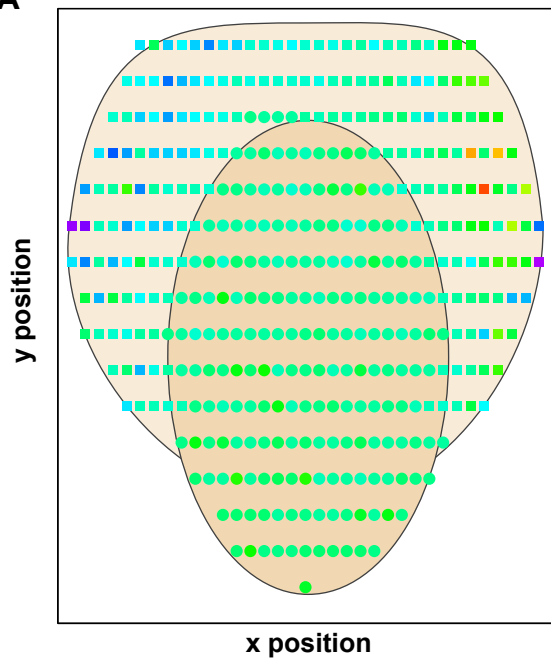**B**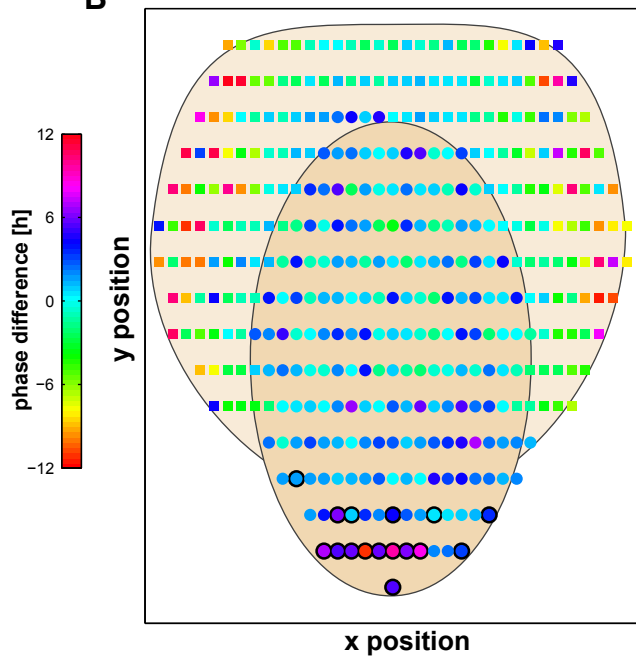**C**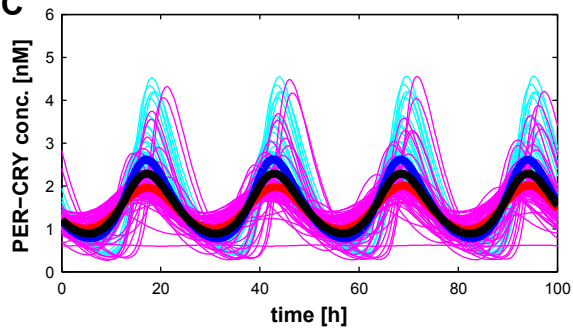**D**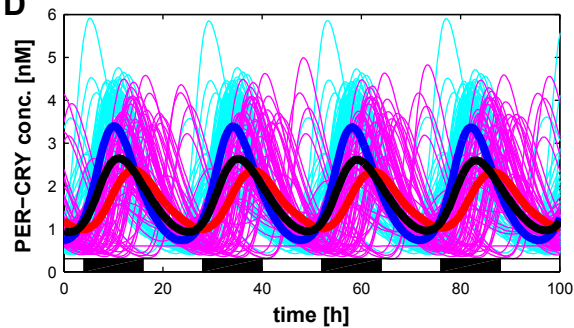

Supplement: Figure S8 — Simulation of the SCN with different architectures of the VL and DM regions (an network coupled to an one, see Fig. S7 for a sketch of the topology) with . (A–B) Phase difference between the cells in DD (A) and LD (B) conditions. Dots represent the cells of the VL (beige region) and squares the DM cells (light yellow region). Green corresponds to a phase delay, blue to a phase advance and red to antiphase. (C–D) Concentration of in the individual VL (cyan lines) and DM cells (magenta lines) and average over the VL (thick blue line) and DM cells (thick red line) and the entire SCN (thick black line) in DD (C) and LD (D) conditions. (PDF) [file pcbi.1002419.s008.pdf]

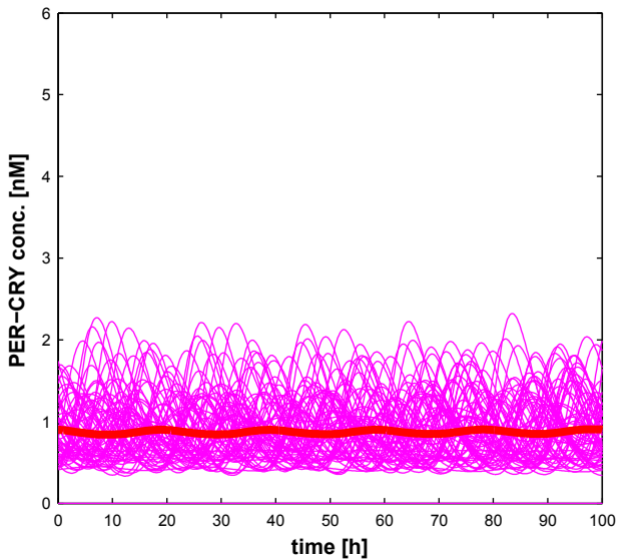

Supplement: Figure S9 — Simulation of the cells of the DM with a coupling constant isolated from the VL cells (corresponding to Fig. 8 of the main text). (PDF) [file pcbi.1002419.s009.pdf]

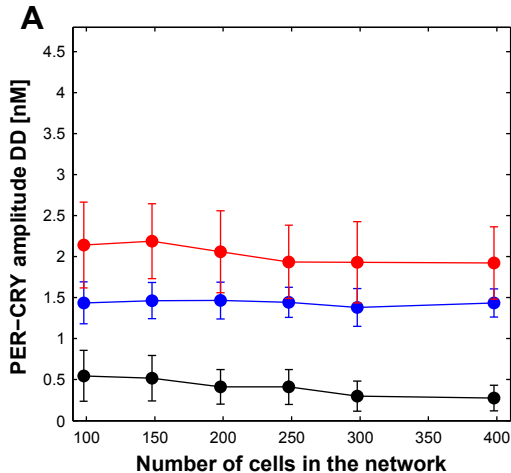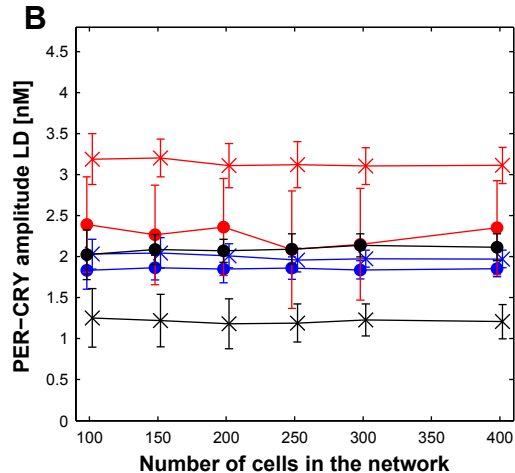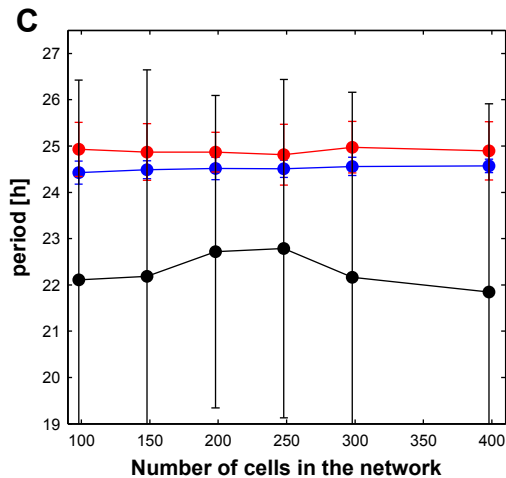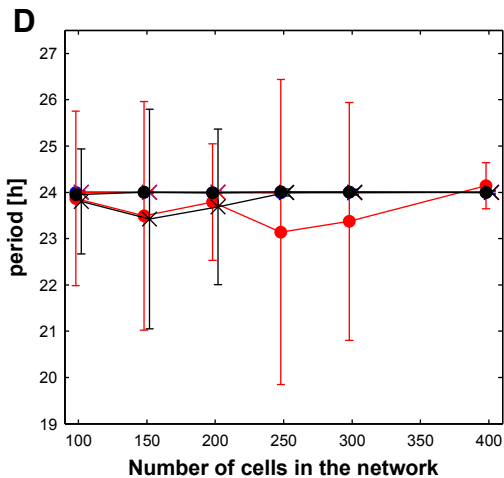

Supplement: Figure S10 — Effect of the number of cells on the network properties. (A–B) Amplitude of the oscillations of in the DD (C) and LD (D) conditions for different network types with and a network size from 100 to 400 cells. (C–D) Period of the oscillations of in the DD (C) and LD (D) conditions for different network types with and a network size from 100 to 400 cells. Both properties are independent of the network size (p-value for correlation with the network size is above 0.05 for all combinations except for the amplitude of the networks in DD). (PDF) [file pcbi.1002419.s010.pdf]

**A**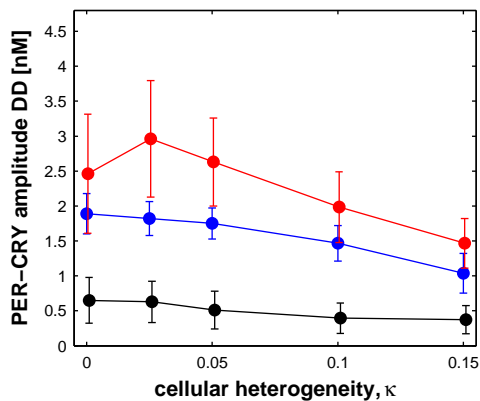**B**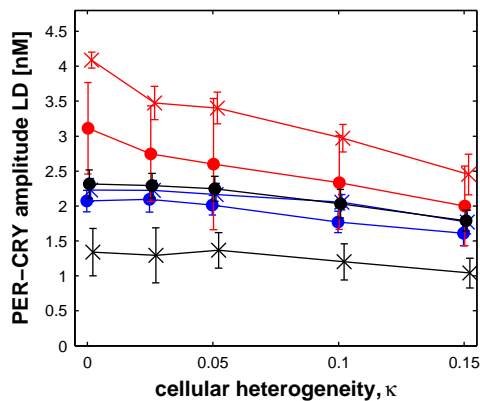**C**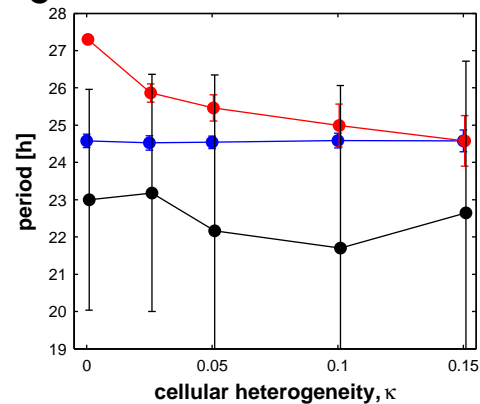**D**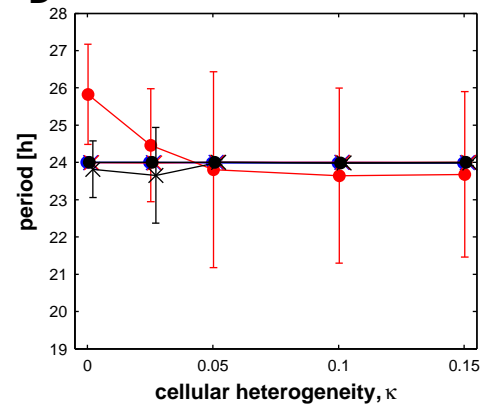**E**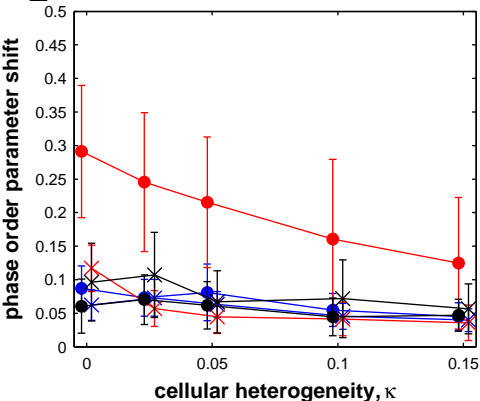**F**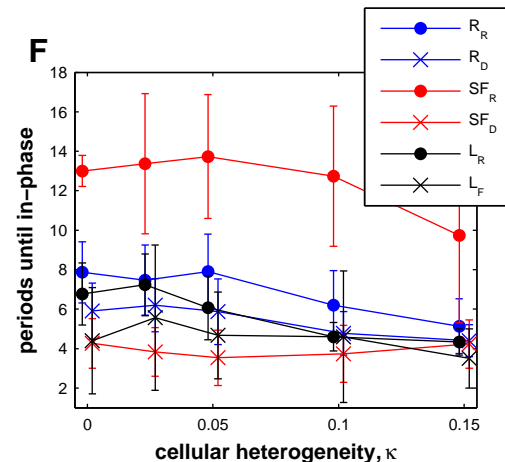

Supplement: Figure S11 — Effect of the cellular heterogeneity parameterized by on the network properties. (A–B) Amplitude of the oscillations of in the DD (C) and LD (D) conditions for different network types with and . In the range the amplitude is almost constant. (C–D) Period of oscillations in the DD (C) and LD (D) conditions for different network types with and . Periods of the and networks are hardly influenced by , whereas networks have a period closer to 24 hours for large values. (E–F) Decrease in the phase order parameter after the jet lag (E) and number of cycles needed for the phase to be within 1 hour of the phase prior to the jet lag (F) for different network types with and . In general, cellular heterogeneity speeds up resynchronization of the network after the perturbation. (PDF) [file pcbi.1002419.s011.pdf]

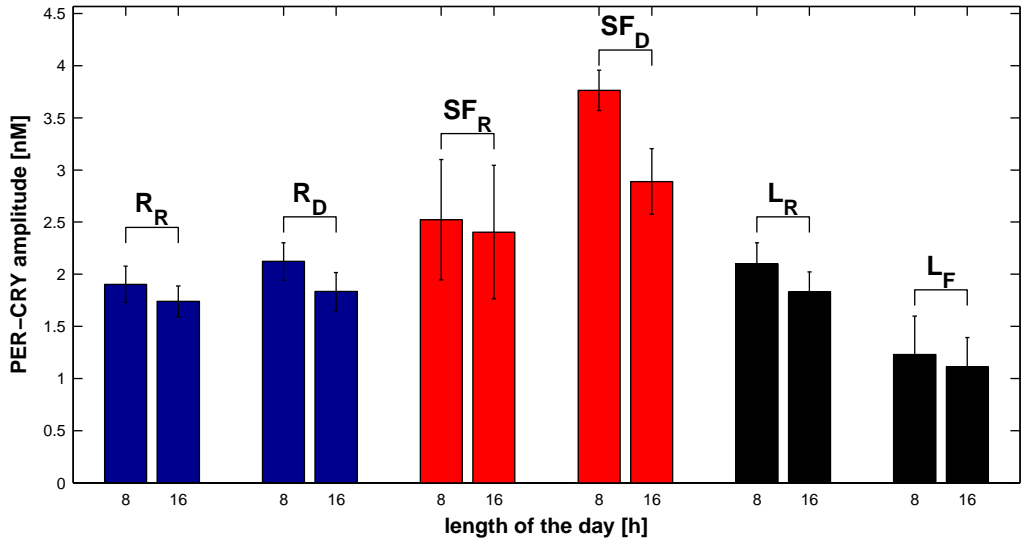

Supplement: Figure S12 — Changes in the oscillation amplitude for short and long days. Amplitude in LD conditions for the six types of networks with either 8 hours (left bar) or 16 hours (right bar) of light (the period of the cycle remains 24 hours). All topologies have ampler oscillations for shorter days, consistent with [49]. (PDF) [file pcbi.1002419.s012.pdf]

**A****DD conditions**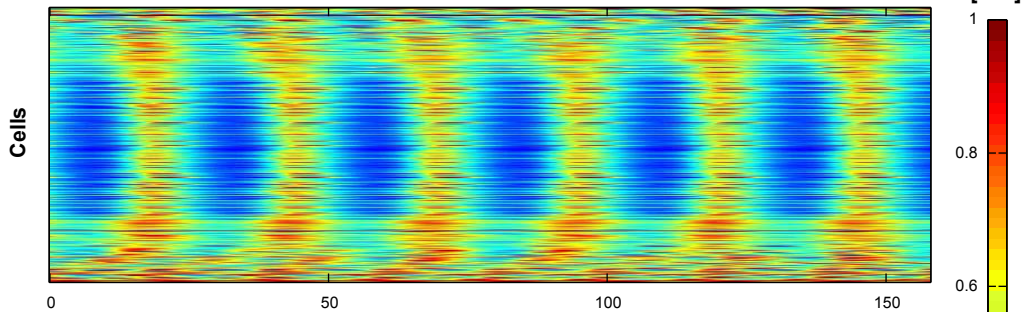**B****LD conditions**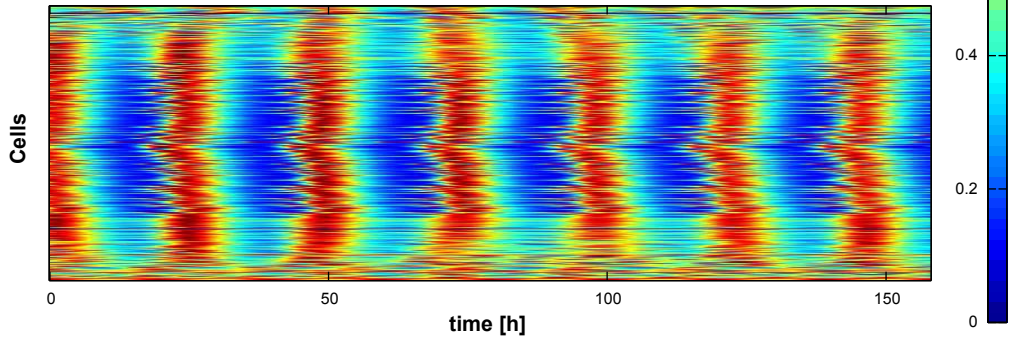

Supplement: Figure S13 — Wave propagation in the combined VL and DM model of the SCN. (A) Normalized concentration in DD conditions as a function of time (y-axis) for the 400 cells (x-axis), corresponding to the network in figures 8 and S7. The cells closest to the optical chiasm (corresponding to the VL) are shown in the middle and the cells furthest (DM cells) are at the edge. (B) Normalized concentration in LD conditions for the same network. (PDF) [file pcbi.1002419.s013.pdf]
