# Supplementary material for: Effect of Network Architecture on Synchronization and Entrainment Properties of the Circadian Oscillations in the Suprachiasmatic Nucleus
Source: PLoS Comput Biol. 2012 Mar 8;8(3):e1002419. doi: 10.1371/journal.pcbi.1002419 (PMC3297560; doi:10.1371/journal.pcbi.1002419)
Supplement: Table S1 — Value and description of the parameters of the model (adapted from [24] ). (PDF) [file pcbi.1002419.s014.pdf]

Parameter	Default value	Description
k_1	6.89 nMh^{-1}	Maximal rate of Per/Cry transcription
k_2	0.90 nM	Michaelis constant of Per/Cry transcription
k_3	0.50 nM	Inhibition constant of Per/Cry transcription
k_4	0.126 h^{-1}	Degradation rate of Per/Cry mRNA
k_5	0.257 nMh^{-2}	Synthesis rate of PER/CRY protein
k_6	0.076 h^{-1}	Degradation rate of cytoplasmatic PER/CRY
k_7	0.248 h^{-1}	Nuclear import rate of the PER/CRY complex
k_8	0.014 h^{-1}	Nuclear export rate of the PER/CRY complex
k_9	0.135 h^{-1}	Degradation rate of the nuclear PER/CRY complex
k_{10}	1.24 nMh^{-1}	Maximal rate of Bmal1 transcription
k_{11}	1.94 nM	Michaelis constant of Bmal1 transcription
k_{12}	0.12 h^{-1}	Degradation rate of Bmal1 mRNA
k_{13}	0.186 h^{-1}	Synthesis rate of BMAL1 protein
k_{14}	1.94 h^{-1}	Degradation rate of cytoplasmatic BMAL1
k_{15}	0.345 h^{-1}	Nuclear import rate of BMAL1
k_{16}	0.046 h^{-1}	Nuclear export rate of BMAL1
k_{17}	0.124 h^{-1}	Degradation rate of nuclear BMAL1
k_{18}	0.069 h^{-1}	Activation rate of nuclear BMAL1
k_{19}	0.0023 h^{-1}	Deactivation rate of nuclear BMAL1*
k_{20}	0.088 h^{-1}	Degradation rate of nuclear BMAL1*
k_{21}	0.076 h^{-1}	Production rate of the neurotransmitter
k_{22}	2.84 h^{-1}	Degradation rate of the neurotransmitter
k_{23}	13.5 nM	Total concentration of PKA
k_{24}	5.4 h^{-1}	Activation rate of PKA
k_{25}	7.2 h^{-1}	Deactivation rate of PKA
k_{26}	13.5 nM	Total concentration of CREB
k_{27}	0.27 h^{-1}	Activation rate of CREB
k_{28}	9.00 h^{-1}	Deactivation rate of CREB
