# Supplementary material for: Effect of Network Architecture on Synchronization and Entrainment Properties of the Circadian Oscillations in the Suprachiasmatic Nucleus
Source: PLoS Comput Biol. 2012 Mar 8;8(3):e1002419. doi: 10.1371/journal.pcbi.1002419 (PMC3297560; doi:10.1371/journal.pcbi.1002419)
Supplement: Table S2 — Statistics of the networks used for results of figure 6 . All values are the average over 30 different networks. For the average degree, the value in parenthesis is the desired value. (PDF) [file pcbi.1002419.s015.pdf]

Network type	Average degree $\bar{d}$ (desired)	Fraction of light-sensitive cells (0.2)	Fraction of cells without incoming edge	Number of weakly connected components
R_R	3.01 (3)	0.20	0.048	1.3
	4.00 (4)	0.20	0.017	1
	5.00 (5)	0.20	0.008	1.07
	6.97 (7)	0.20	0.001	1
	9.05 (9)	0.20	0.000	1
	12.05 (12)	0.20	0	1
	14.99 (15)	0.20	0	1
R_D	3.00 (3)	0.20	0.052	1.6
	4.05 (4)	0.20	0.016	1
	5.04 (5)	0.19	0.007	1.07
	6.92 (7)	0.19	0.001	1
	8.97 (9)	0.20	0.000	1
	11.96 (12)	0.20	0	1
	14.91 (15)	0.20	0	1
SF_R	3 (3.00)	0.20	0	1
	4.00 (4)	0.19	0	1
	5.00 (5)	0.21	0	1
	7.00 (7)	0.20	0	1
	9.00 (9)	0.20	0	1
	12.00 (12)	0.19	0	1
	15.00 (15)	0.19	0	1
SF_D	3 (3.00)	0.19	0	1
	4.00 (4)	0.19	0	1
	5.00 (5)	0.20	0	1
	7.00 (7)	0.19	0	1
	9.00 (9)	0.20	0	1
	12.00 (12)	0.19	0	1
	15.00 (15)	0.19	0	1
L_R	3 (2.99)	0.21	0.022	1.1
	4.02 (4)	0.21	0.003	1
	4.86 (5)	0.19	0	1
	6.92 (7)	0.19	0	1
	8.93 (9)	0.20	0	1
	11.95 (12)	0.20	0	1
	14.95 (15)	0.20	0	1
L_F	3 (3.01)	0.20	0.017	1.1
	3.99 (4)	0.20	0.003	1
	4.87 (5)	0.20	0	1
	6.89 (7)	0.20	0	1
	8.91 (9)	0.20	0	1
	11.93 (12)	0.20	0	1
	14.94 (15)	0.21	0	1
